# Validity Assessment of Wrist-Worn Electrodermal Activity Measurement During Relaxation and Acute Stressor Tasks: Comparing the EmbracePlus and Shimmer3 GSR+

**DOI:** 10.3390/s26216795

**Published:** 2026-10-27

**Authors:** Manon W. H. Peeters, Bo A. C. Dirks, Sjoerd Kesteloo, Bart van Boxtel, Petra Heck, Gerard Schouten

**Affiliations:** 1 School for Allied Health Professions, Fontys University of Applied Sciences, Postbus 347, 5600 AH Eindhoven, The Netherlandsbart.vanboxtel@fontys.nl (B.v.B.); 2 Human-Technology Interaction Group, Faculty Industrial Engineering & Innovation Sciences, Eindhoven University of Technology, Postbus 513, 5600 MB Eindhoven, The Netherlands; 3 School for Information & Communication Technology, Fontys University of Applied Sciences, Postbus 347, 5600 AH Eindhoven, The Netherlands; p.heck@fontys.nl (P.H.); g.schouten@fontys.nl (G.S.)

**Keywords:** electrodermal activity, wearable validation, EmbracePlus, Shimmer3 GSR+, stress

## Abstract

Wearable electrodermal activity (EDA) monitoring enables assessment of stress-related physiological changes in daily life, but new devices require validation. This study evaluated the newly introduced EmbracePlus against the Shimmer3 GSR+, used as a research-grade wrist-based comparator. Healthy volunteers (*n* = 53; *n* = 41 valid paired recordings) completed breathing exercises, a Stroop task, and a virtual reality (VR) height stressor while wearing both devices simultaneously on the non-dominant wrist. Agreement was assessed at signal, parameter, and event levels for mean phasic activity, skin conductance level (SCL), summed skin conductance response (SCR) amplitude/min, and SCR count/min. Mean signal-level cross-correlation was r = 0.30. At the parameter level, only 44% of SCL, 24% of SCR amplitude/min, and 5% of SCR count/min differences fell within a priori acceptable boundaries, and regression analyses indicated proportional bias for mean phasic activity, summed SCR amplitude/min and SCR count/min. At the event level, Shimmer3 detected increases across all four EDA metrics during both stress tasks (all *p* < 0.001). EmbracePlus showed increases across all four metrics during VR (all *p* < 0.001), whereas during Stroop only mean phasic activity (*p* = 0.001) and SCL (*p* < 0.001) increased. EmbracePlus and Shimmer3 are therefore not interchangeable for absolute EDA assessment, and EmbracePlus validity depends on stressor intensity and metric selection.

## 1. Introduction

Stress is a complex phenomenon that plays a major role in both physical and mental health [1]. The term stress is used to describe multiple related processes, including exposure to stressors and the psychological and physiological responses they elicit [2,3]. Physiological stress responses involve activation of multiple systems, including the autonomic nervous system [4], resulting in measurable physiological responses, including changes in cardiac activity, skin temperature, and sweat gland activity [5]. Electrodermal activity (EDA), which reflects sweat gland activity under sympathetic control, is therefore frequently used to assess physiological reactivity to stressors [5,6].

**Figure 1:**
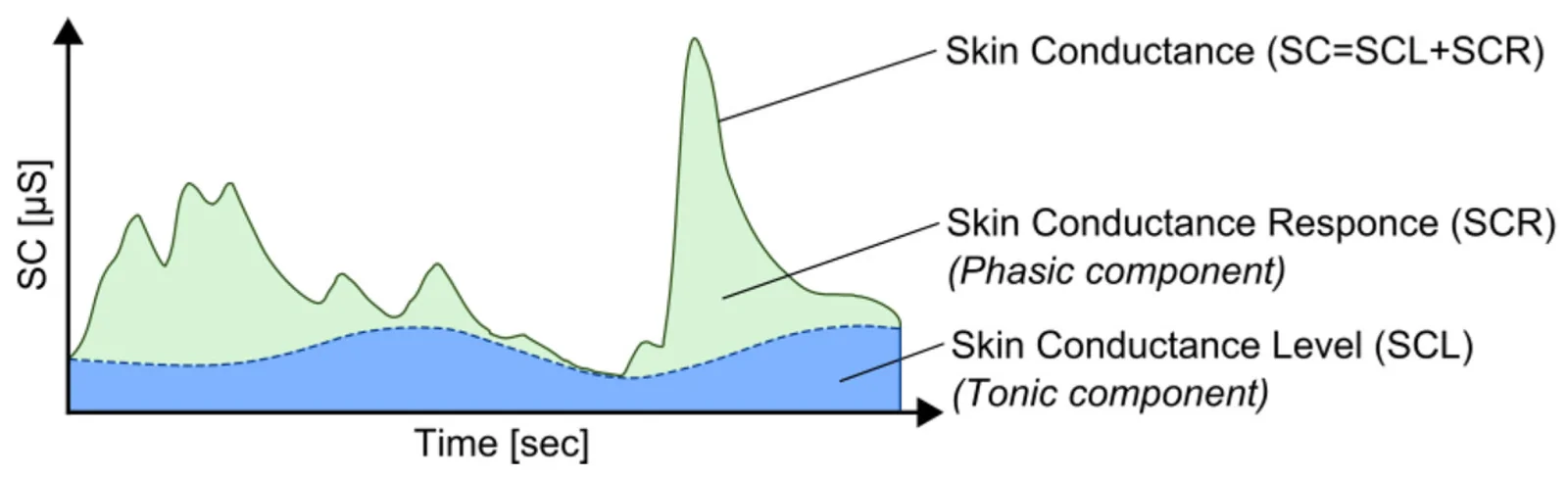
The tonic and the phasic components of skin conductance (SC) [8]. CC-BY-NC.

EDA can be measured through changes in skin conductance (SC), which can be differentiated into tonic and phasic components [6] (see Figure 1). The tonic component, commonly expressed as the skin conductance level (SCL), reflects slower changes in skin conductance and is associated with more sustained changes in general arousal. In contrast, the phasic component consists of more rapid skin conductance responses (SCRs), which reflect transient responses to stimuli and can be influenced by stimulus novelty, intensity, and psychological significance [6]. Phasic activity can be quantified by characteristics such as SCR frequency and amplitude, while area-based measures provide an integrated measure of phasic activity over time [6]. Because tonic and phasic EDA capture different aspects of electrodermal activation, and SCR frequency and amplitude do not necessarily covary [7], assessing SCL together with multiple phasic measures provides complementary information on stress-related EDA responses.

Wearable sensors increasingly facilitate continuous, non-invasive monitoring of EDA, both in controlled laboratory settings and in everyday contexts [9]. Recent work on physiological workload monitoring identifies EDA as a frequently used modality in wearable and multimodal assessment of cognitive and physical demands [10]. Wrist-based EDA is particularly suited to prolonged ambulatory monitoring because it can be incorporated into wearable devices suitable for daily life recordings [11,12]. However, palmar and volar finger sites remain the conventional locations for psychophysiological EDA recording, and wrist EDA may additionally be influenced by thermoregulatory sweating [6,7]. Absolute EDA values and response characteristics at the wrist may therefore differ from conventional palmar recordings. Nevertheless, wrist-recorded EDA has been shown to respond to experimentally induced changes in sympathetic activity, supporting its relevance for wearable monitoring [11]. Continuous and ambulatory monitoring of stress-related physiological responses has the potential to provide insight into daily fluctuations, support early detection of maladaptive stress responses, and inform personalized interventions in both research and healthcare [9]. For such applications, however, wearable EDA measurements need to be validated in a transparent and reproducible manner. Validation studies differ in the levels of analysis, statistical approaches, and criteria used to evaluate device performance, which can limit the interpretability and comparability of findings across studies. Wearable devices differ substantially in electrode placement, signal quality, data accessibility, and degree of validation [12,13]. Such variability complicates the scientific interpretability and comparability of findings across studies. These challenges highlight the need for continuous and reproducible validation, particularly when established devices are replaced by newer ones, as evidence from past validation studies does not necessarily transfer to their successors.

In stress research, the Empatica E4 (Empatica Inc., Milan, Italy) has been widely adopted and is among the most frequently used devices for wearable EDA measurement. It has been applied in both clinical and experimental studies and evaluated in several validation efforts [9,13]. However, the Empatica E4 was discontinued in 2025, and Empatica introduced the EmbracePlus as its successor. The EmbracePlus is CE/MDR certified and, for EDA measurement, uses dry electrodes at the wrist, making it relevant for evaluation in applied and clinical contexts. The EmbracePlus has already been adopted in applied research contexts, for instance, to detect agitation in dementia patients [14], to evaluate music-based biofeedback interventions for stress reduction in people with mild intellectual disabilities [15], and to evaluate heart rate accuracy alongside other wearables [16]. These applications demonstrate the practical relevance of the EmbracePlus, but do not by themselves establish how its EDA measurements compare with those obtained simultaneously from a research-grade comparator. Initial comparative work further suggests that the EmbracePlus can capture stress-related EDA responses relative to a medical-grade reference system, although this work has primarily focused on signal fidelity, peak detection, and artifact behavior rather than on a broader validation of derived EDA metrics [17]. More recently, an exploratory comparison with the Empatica E4 [18] found lower absolute EDA values for the EmbracePlus, while relative task-related patterns were broadly comparable between devices. However, this comparison was based on separate participant cohorts rather than simultaneous recordings, limiting conclusions about direct device agreement. Acan et al. [19] subsequently compared the EmbracePlus and Empatica E4 during concurrent recordings and found stronger correspondence for phasic than tonic EDA features, together with systematically lower absolute EDA amplitudes for the EmbracePlus. This provides important evidence on cross-device consistency. Acan et al. [19] primarily focused on synchronization, interoperability, and signal- and feature-level agreement during prolonged recordings rather than on device performance during controlled stress induction.

Demonstrating that a wearable detects expected stress-related physiological changes provides evidence relevant to construct validity, whereas correspondence between independent measurement methods intended to assess the same physiological signal provides evidence relevant to convergent validity [20,21]. These complementary sources of validity evidence are important because the ability to detect expected physiological changes does not necessarily imply that measurements correspond with those obtained using another measurement system. Such validation of wearable measurements is crucial, since the choice of device strongly influences measurement quality, and differences in electrode type, placement, and signal processing can substantially influence measurement outcomes [12,13], while differences in validation approaches may further limit the interpretability and comparability of findings across studies.

To address these challenges and improve the consistency, interpretability, and comparability of wearable validation studies, Van Lier et al. [13] proposed a standardized multi-level validity assessment framework. The framework distinguishes three levels of comparison: the signal level, which evaluates similarity between the raw physiological signals; the parameter level, which evaluates agreement in physiological parameters aggregated over a defined time frame for each participant; and the event level, which evaluates whether physiological changes associated with events or phenomena of interest can be detected by both devices. Evaluating these levels separately allows device agreement at the raw-signal and parameter levels to be distinguished from the ability to detect physiologically relevant changes in the intended context of use. Together, the available evidence indicates that comparisons of EmbracePlus EDA measurements with those obtained from other EDA devices have begun to emerge, but systematic validation during controlled stress induction and across complementary signal, parameter, and event levels remains limited.

Given the intended use of the EmbracePlus for ambulatory monitoring, this study specifically focuses on wrist-worn EDA measurement. The Shimmer3 GSR+ (Shimmer Research, Dublin, Ireland) was selected as a research-grade comparator and has been applied in experimental and wearable-comparison studies [22,23]. The system allows EDA acquisition using externally positioned electrodes, including pre-gelled Ag/AgCl electrodes as used in previous experimental research [24]. Its flexible electrode configuration has also been used to acquire EDA at different body sites [25]. This allowed the electrodes in the present study to be positioned on the same wrist as the EmbracePlus, enabling simultaneous within-participant comparison while minimizing differences attributable to anatomical measurement site. Therefore, the present study aims to systematically compare wrist-worn EDA measurements obtained with the EmbracePlus and the Shimmer3 GSR+ and to evaluate evidence relevant to the validity of the EmbracePlus across the signal, parameter, and event levels of the multi-level framework proposed by Van Lier et al. [13].

## 2. Materials and Methods

### 2.1. Participants

Healthy volunteers (*n* = 53; 29 males, 24 females; mean age = 30.1 years, SD = 11.8) were included in this study. Participants were recruited via convenience sampling among students, employees, and affiliates of Fontys University of Applied Sciences in Eindhoven, Netherlands. Eligibility criteria included being at least 16 years of age, able to communicate in Dutch or English, and free of conditions or medications that could influence stress reactivity in our study (e.g., beta-blockers, acrophobia, balance problems, or color blindness).

### 2.2. Ethical Considerations

The study was conducted in accordance with the ethical guidelines of the Declaration of Helsinki. The Fontys Committee of Research Ethics approved the study (approval code “peeters-schaap250325”). All participants provided written informed consent. Participation was voluntary, with the right to withdraw at any time. All data were anonymized and handled in accordance with the European General Data Protection Regulation (GDPR).

### 2.3. Measuring Instruments

#### 2.3.1. Physiological Measurements

**Figure 2:**
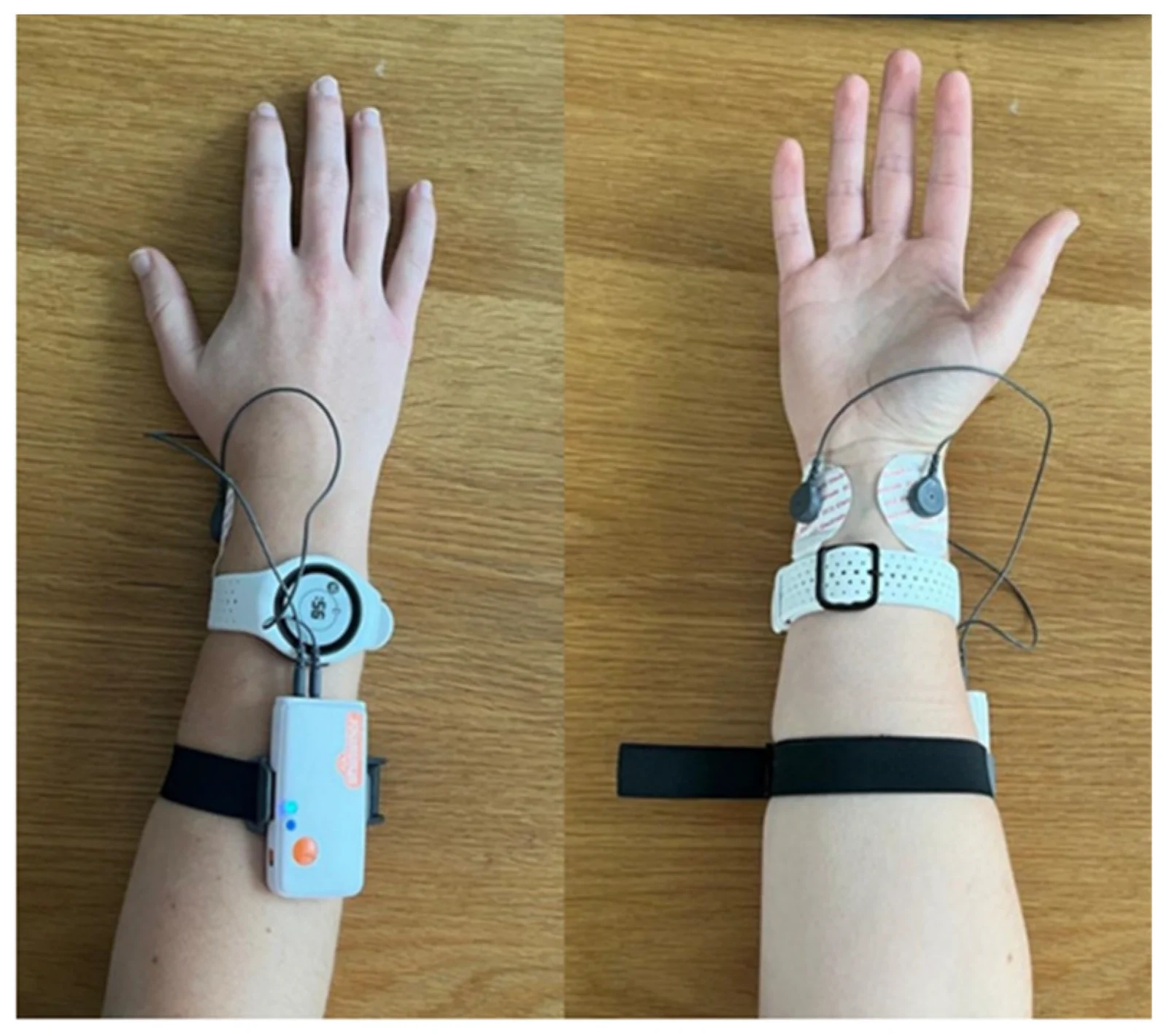
Placement of the wearable devices. The EmbracePlus (wristband) and Shimmer3 GSR+ (sensor with electrode stickers) devices were worn on the non-dominant wrist.

EDA was measured wirelessly using two wearable devices: The EmbracePlus (Empatica Inc., Milan, Italy), a CE-marked and MDR Class 2A registered wrist-worn device, using dry electrodes on the ventral side of the wrist. Secondly, the Shimmer3 GSR+ Unit (Shimmer Sensing, Dublin, Ireland), CE-marked and research-grade, was used with pre-gelled Ag/AgCl electrodes. This configuration was selected to provide a stable electrode–skin contact during the laboratory measurements [6]. Both devices were worn simultaneously on the non-dominant wrist (see Figure 2). The EmbracePlus was placed in its default wristwatch position, while the Shimmer3 GSR+ electrodes were placed distally on the same wrist, approximately 1 cm apart from the EmbracePlus electrodes to avoid signal interference. Both devices recorded EDA at a sampling rate of 4 Hz throughout the entire session. EDA data from the EmbracePlus were collected using the Care Lab application by Empatica (version 5.56.4), while data from the Shimmer3 GSR+ were acquired using the free version of ConsensysBasic software (Shimmer Sensing, Dublin, Ireland, version 1.6.0).

#### 2.3.2. Subjective Perceived Stress

**Figure 3:**
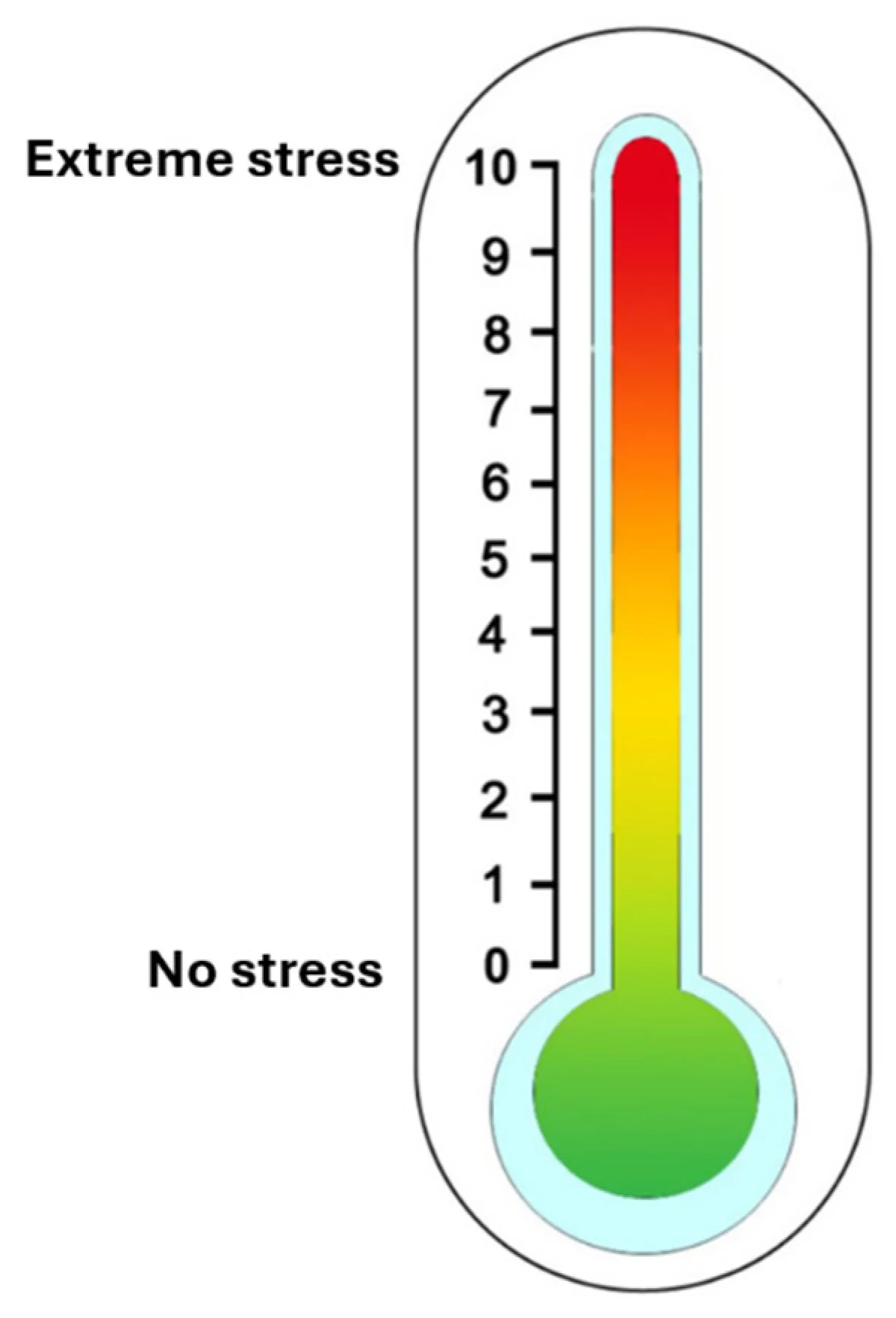
The stress thermometer used to ask participants how stressful a task was experienced. Adapted from Nixon et al. [26], licensed under CC BY 4.0.

Self-reported perceived stress was assessed after each task (excluding the baseline) using a visual numeric rating scale shown as a *stress thermometer* printed on paper (see Figure 3). Participants were asked, “How stressed did you feel during this task?” and instructed to respond with a number from 0 (*not at all stressed*) to 10 (*extremely stressed*). Responses were communicated verbally by participants and written down by the researcher.

### 2.4. Procedure

**Figure 4:**
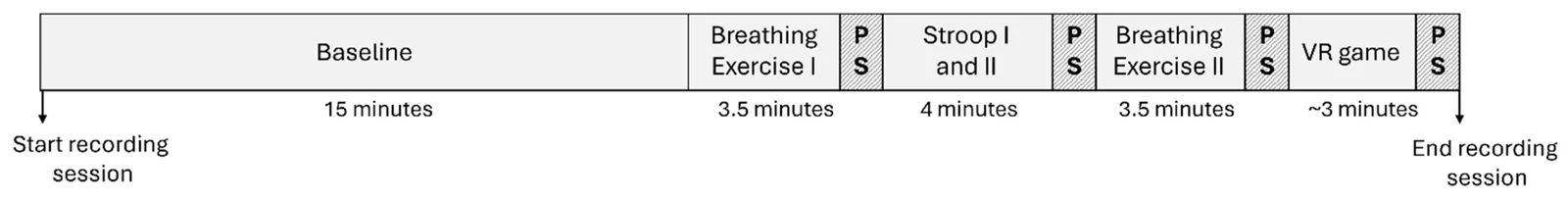
Timeline of the study. PS indicates perceived stress assessment.

All participants were tested individually in a quiet, temperature-controlled lab setting. After informed consent and a short demographic questionnaire (i.e., gender and age), the ventral side of the non-dominant wrist was cleaned with 70% isopropyl alcohol using Comed Alcomed Pads (Strasbourg, France), and both the EmbracePlus and Shimmer3 GSR+ devices were placed (see Figure 2). Two trained researchers remained in the room while conducting the experiment, observing and logging data. Each session followed a fixed timeline of approximately 35 min (see Figure 4). The Stroop and VR tasks were selected to represent complementary acute stressor contexts [21]: cognitive performance stress during the Stroop task [27,28] and threat/fear-related arousal during virtual height exposure [29]. Both experimental stressors were expected to elicit increased EDA [27,28,29]. Task order was fixed, and no randomization was applied. The Stroop task preceded the VR height stressor, with a breathing exercise before each stressor, to progress from a cognitive performance stressor to a more immersive threat/fear-related stressor and to reduce potential carryover from the VR condition into the Stroop task.

1. Start recording session

Participants were seated in a relaxed upright position. The main instruction was: *“Please sit still and relax for the next 15 min”.* During this phase, participants received instructions and/or answers to their questions regarding the experiment. This baseline period served to provide good contact between the electrodes and the skin, especially for the EmbracePlus’ dry electrodes.

2. Breathing Exercise 1 (3.5 min)

The experimenter sat nearby and verbally guided the participant through repetitions of the 4-7-8 breathing technique [30]. The timing of inhalation (4 s), holding (7 s), and exhalation (8 s) was counted aloud, with a short pause before each new repetition. The total duration of the exercise was approximately 3.5 min. Participants were informed that they could choose whether to keep their eyes open or closed during the exercise.

3. Stroop Task (twice; 4 min)

A computerized version of the Stroop Color-Word Task [31] was presented on a laptop using a custom-built PowerPoint slideshow in full-screen mode. Each slide requiring a verbal response was displayed for 0.8 s. To enhance performance pressure and cognitive task demands, participants were encouraged to achieve a high score, and the number of correct and incorrect responses was monitored. The task consisted of four sequential phases. In the first phase, participants were shown 20 slides containing a single color word (e.g., “red”) in black font. They were instructed to read each word aloud. In the second phase, another 20 slides were presented, this time showing only solid background colors without any text. Participants were instructed to name the displayed color aloud. In the third phase, 10 slides were shown in which the color of the text was incongruent with the written word (e.g., “yellow” written in blue ink). Participants were instructed to name the ink color of the word, rather than reading the word itself, and to respond as quickly and accurately as possible. The fourth and final phase included 11 slides that presented a color word in incongruent ink color on a mismatching background color, further increasing the complexity of the task. After approximately 15 s, participants were instructed to repeat the Stroop task with the explicit instruction to improve their score compared to the first attempt.

4. Breathing Exercise 2 (3.5 min)

Identical to Breathing Exercise 1.

5. Stressor 2—Virtual Reality (3 min)

Participants wore a Meta Quest 1 VR headset (Meta Platforms Inc., Menlo Park, CA, USA) and engaged with the immersive game Richie’s Plank Experience (Toast VR, Gold Coast, QLD, Australia). In this scenario, participants were instructed to enter a virtual elevator, ascend to a high floor (80 stories high), and walk out onto a narrow plank suspended above a virtual city. To enhance realism and embodiment, a physical wooden plank (180 × 14.5 cm) was placed on the laboratory floor and carefully calibrated to match the virtual plank’s dimensions and position. Participants were encouraged to physically walk onto the plank while immersed in the VR environment, inducing a height illusion intended to elicit a stress response. They were given the option to “jump” off the virtual plank if desired. After walking the plank once or jumping, they were asked whether they wanted to take the virtual elevator down (in the case of non-jumping) and repeat the task.

The researcher remained nearby throughout the condition to ensure safety, provide headset support, and verbally guide the participant when needed. The VR experience was simultaneously cast to a tablet, allowing the researcher to monitor the participant’s field of view and offer real-time instructions or reassurance as necessary.

After Breathing Exercise I, the second Stroop, Breathing Exercise II, and VR, participants verbally rated their perceived stress on a 0–10 scale using the stress thermometer (Figure 3).

### 2.5. Data Synchronization

The physiological recordings were synchronized using the device-generated UNIX timestamps. To ensure alignment between the active conditions and the physiological data, all key protocol events (e.g., the start and end of each condition) were manually time-stamped using the local system time format (hh:mm:ss) in a dedicated observation Excel sheet. A Visual Basic for Applications (VBA) macro button was used to record timestamps in real time. These system time logs were later aligned with the UNIX timestamps embedded in the physiological data files exported from Care Lab (Empatica) and ConsensysBasic (Shimmer), enabling alignment of the physiological recordings with the observational timeline. The one-second event-marker resolution was considered adequate because the present analyses were performed on complete condition-level segments rather than on stimulus-locked responses.

### 2.6. Data Processing and Analysis

#### 2.6.1. Perceived Stress

Analysis of perceived stress scores was conducted using IBM SPSS Statistics version 29. To ensure direct comparability with the physiological analyses, perceived stress was analyzed only for the participants retained for physiological signal analysis (*n* = 41; see Physiological data section for exclusion procedure). To examine whether perceived stress levels differed significantly across the four conditions (Breathing Exercise I, Stroop, Breathing Exercise II, and VR), a non-parametric Friedman test was performed. This test was chosen due to the within-subjects design and the ordinal nature of the stress ratings. Additionally, QQ plots indicated violations of the normality assumption. When the Friedman test revealed a statistically significant main effect, Kendall’s W was calculated as an effect size [32]. Subsequently, post hoc pairwise comparisons were conducted using the Wilcoxon signed-rank test with effect sizes expressed as r [32]. To control for multiple comparisons, a Bonferroni correction was applied, adjusting the significance threshold to α = 0.0083 (i.e., 0.05 divided by 6 comparisons).

#### 2.6.2. Physiological Data and Pre-Processing

EDA was recorded in micro siemens at 4 Hz with UNIX timestamps, and either exported as AVRO files from the Empatica CareLab environment (EmbracePlus) or as CSV files from the local Consensys program (Shimmer3 GSR+). Using custom-developed software in Python 3.12, both AVRO [33] and CSV files were converted to Excel format with human-readable timestamps. Subsequently, time-stamped experimental events (i.e., 15 min baseline, breathing exercise I, Stroop I and II, breathing exercise II and VR) were manually added to these Excel files, resulting in labeled EDA datasets.

All raw EDA signals (*n* = 106, as each participant has two signals) were visually inspected by two researchers. Signals with physiologically impossible values or patterns (e.g., sudden drops to 0, EDA values below 0), indicating skin contact issues or issues with the sensor, were excluded from further analysis. Participants 4, 11–12, 15, 27, 34–36, 43, 46, and 52–53 were therefore excluded, leading to 41 included participants. Of these 12 exclusions, five were due to EmbracePlus signal-quality issues (participants 4, 15, 36, 43, and 46) and seven were due to Shimmer3 GSR+ signal-quality issues (participants 11, 12, 27, 34, 35, 52, and 53). No participant was excluded because of signal-quality issues in both devices. Participants 10, 33, 48 and 50 showed values < 0.01 µS during a small part of their baseline recordings; these segments were excluded during preprocessing (i.e., their signal between the start of the signal and Breathing Exercise 1 was shortened), while the remaining signal was retained for analysis.

For the validity assessment, the three-level framework introduced by Van Lier et al. [13] was operationalized through separate signal-, parameter-, and event-level analyses, as described below. The three levels were considered complementary rather than interchangeable, with each addressing a different aspect of device performance.

#### 2.6.3. Signal-Level Agreement

To evaluate the similarity between the raw EDA signals from both devices, a lagged Pearson cross-correlation analysis was performed in Python version 3.12, using NumPy (version 2.2.4) and SciPy (version 1.15.2). First, signals were normalized (z-scored) and linearly detrended. For each participant, Pearson correlation coefficients were calculated across lags ranging from −4 to +4 data points (i.e., ±1 s) to account for potential temporal misalignments, and the maximum correlation coefficient was retained. In addition to the full-signal analysis, a secondary segment-specific signal-level analysis was performed for the interval from the start of Breathing Exercise I to the end of Breathing Exercise II, as participants remained seated during this period and relatively few movement artifacts were expected.

#### 2.6.4. Parameter-Level Agreement

Raw EDA (µS) was first screened for short artefact spikes using a z-score–based jump–return rule in Python 3.12 using NumPy (version 2.2.4) and SciPy (version 1.15.2). The code can be found in Supplementary File S1. A sample was marked as an artefact onset when:

(1) the instantaneous deviation from a local baseline (mean of the last 4 non-artefact samples) was ≥0.75 z (“jump”), and
(2) there was a return within 1.5 s in the opposite direction with magnitude ≥ 0.675 z.

All samples from jump to return were flagged as artefact, as these are physiologically highly unlikely to be an SCR [6]. For every detected artefact, we expanded the mask by ± 1.0 s (to treat the immediate surroundings as unreliable). All flagged samples were then linearly interpolated on the raw µS signal using a left anchor (value of the last clean sample before the artifact block) and the first clean sample after the block as the right anchor; if only one anchor was available, the entire block was filled flat with that anchor value. All corrected signals underwent visual inspection by two researchers.

Subsequently, signals were segmented into contiguous marker-defined segments (i.e., baseline, breathing exercise I, Stroop I and II, breathing exercise II, VR and undefined periods), and processed with NeuroKit2 (version 0.2.10) in Python 3.12 [34]. We extracted the tonic and phasic components using cvxEDA [35]. SCR peaks were then identified on the phasic signal using NeuroKit’s prominence-based peak detection with a near-zero relative threshold, after which only peaks with an absolute amplitude of at least 0.01 µS (peak minus onset) were retained [6,7,13]. To capture complementary tonic and phasic aspects of EDA, mean SCL was selected as a tonic measure, while mean phasic activity, summed SCR amplitude, and SCR count were included as complementary phasic measures [6,7]. Mean phasic activity was calculated as the mean of the cvxEDA-derived phasic component over the analyzed interval. Its inclusion provided a continuous measure of phasic activity alongside the peak-based SCR measures, without relying on the detection of individual SCR peaks [36]. From these processed signals, we derived four participant-level summary metrics for device comparison:

1. Mean phasic: mean phasic signal across the analyzed interval (µS);
2. Mean SCL: mean tonic skin conductance across the analyzed interval (µS);
3. Summed SCR amplitude/minute: sum of detected SCR amplitudes normalized per minute (µS/min);
4. SCR count/minute: number of detected SCRs per minute.

Consistent with the parameter-level definition of Van Lier et al. [13], each EDA metric was summarized as one aggregated value per participant and per device over the complete retained recording, including the marker-defined experimental conditions and intervening undefined periods. To assess absolute agreement between devices, we performed Bland–Altman analyses [37] on participant-level summary metrics using IBM SPSS Statistics version 29. For each participant, we computed the mean of the two devices (*x*-axis) and the inter-device difference (Shimmer3 GSR+ minus EmbracePlus; *y*-axis). Systematic bias was quantified as the mean difference across participants (bias), and random variation as the 95% limits of agreement (LoA), defined as bias ± 1.96SD of the differences. Bland–Altman plots were inspected for patterns consistent with proportional bias (mean-dependent differences) and/or heteroscedasticity (mean-dependent variability of differences) and for isolated extreme observations. Distributions of the inter-device differences on the original and LN(x + 1)-transformed scales are provided in Supplementary File S2.

Following the validity-assessment framework of Van Lier et al., we adopted a priori acceptable boundaries, reflecting predefined maximum tolerable inter-device differences of ±1.6 µS for mean SCL, ±0.6 µS for summed SCR amplitude/minute, and ±2.5 SCR count/min [13]. Agreement relative to these predefined boundaries was evaluated on the original measurement scale; this proportion-within-boundaries approach does not require normally distributed inter-device differences [37]. We did not define a priori acceptable boundaries for the mean phasic, as no established physiologically or clinically justified a priori acceptability criteria are available for this metric.

Potential proportional bias was further examined using linear regression of the inter-device difference on the mean of both devices. Because residual normality and homoscedasticity were not consistently met, inference was based on bias-corrected and accelerated (BCa) bootstrap 95% confidence intervals [38,39] derived from 5000 resamples. Sensitivity analyses were performed by repeating the regressions after excluding the observation with the highest Cook’s distance when this value exceeded 1. Analyses were also repeated after LN(x + 1) transformation of the device-specific values [6] to assess the influence of skewness and magnitude-dependent variability. The original scale was retained as primary to preserve interpretation of absolute inter-device differences and comparison with the predefined acceptability boundaries; transformed analyses are reported in the Supplementary Materials.

#### 2.6.5. Event-Level Agreement

At the event level, we assessed whether both devices detected physiological changes across the predefined experimental conditions, following Van Lier et al. [13]. Event-level metrics were derived using the same preprocessing steps as in the parameter-level analyses, but were calculated separately for the predefined experimental conditions: Breathing Exercise 1, Stroop I, Stroop II, Breathing Exercise 2, and VR (see Figure 4). Event-level agreement was assessed by (i) visualizing device responses per event (mean ± SE across participants) and (ii) examining the corresponding inter-device difference scores within each event block (Shimmer3 GSR+ minus EmbracePlus). Differences were summarized descriptively by the event type, and we evaluated whether inter-device discrepancies varied across events, consistent with context-dependent performance differences.

To test whether each device could distinguish between experimental conditions at the event level, we conducted non-parametric within-subject tests due to non-normality of the event-level distributions using IBM SPSS Statistics version 29. Specifically, for each outcome (mean phasic, mean SCL, summed SCR amplitude/min and SCR count/min), we ran separate Friedman tests (k = 3; Breathing exercise 1, Stroop I, Stroop II) for Shimmer3 and for EmbracePlus. For the Breathing exercise 2—VR contrast (k = 2), we used paired-samples Wilcoxon signed-rank tests, again conducted separately for Shimmer3 GSR+ and EmbracePlus. If appropriate, post hoc pairwise comparisons were conducted using Bonferroni correction to control the family-wise error rate at 0.05.

Difference plots were constructed in Microsoft Excel for Microsoft 365 (version 2605). Differences were summarized descriptively per event, and we evaluated whether inter-device discrepancies varied across events, consistent with context-dependent performance differences as described in van Lier et al. [13]. A priori boundaries were set to ±|reference effect|, with the reference effect defined as the average within-subject change from the most recent baseline-to-stressor change (i.e., the true effect) measured by the Shimmer3 GSR+. For the Stroop event, this reference effect was defined as the change from Breathing Exercise 1 to Stroop 1. For the VR event, it was defined as the change from Breathing Exercise 2 to VR.

## 3. Results

### 3.1. Perceived Stress Ratings During Conditions

Friedman’s test revealed a statistically significant difference in perceived stress ratings across conditions, χ^2^(df = 3, N = 41) = 105.041, *p* < 0.001, with a large effect size (Kendall’s W = 0.854).

**Figure 5:**
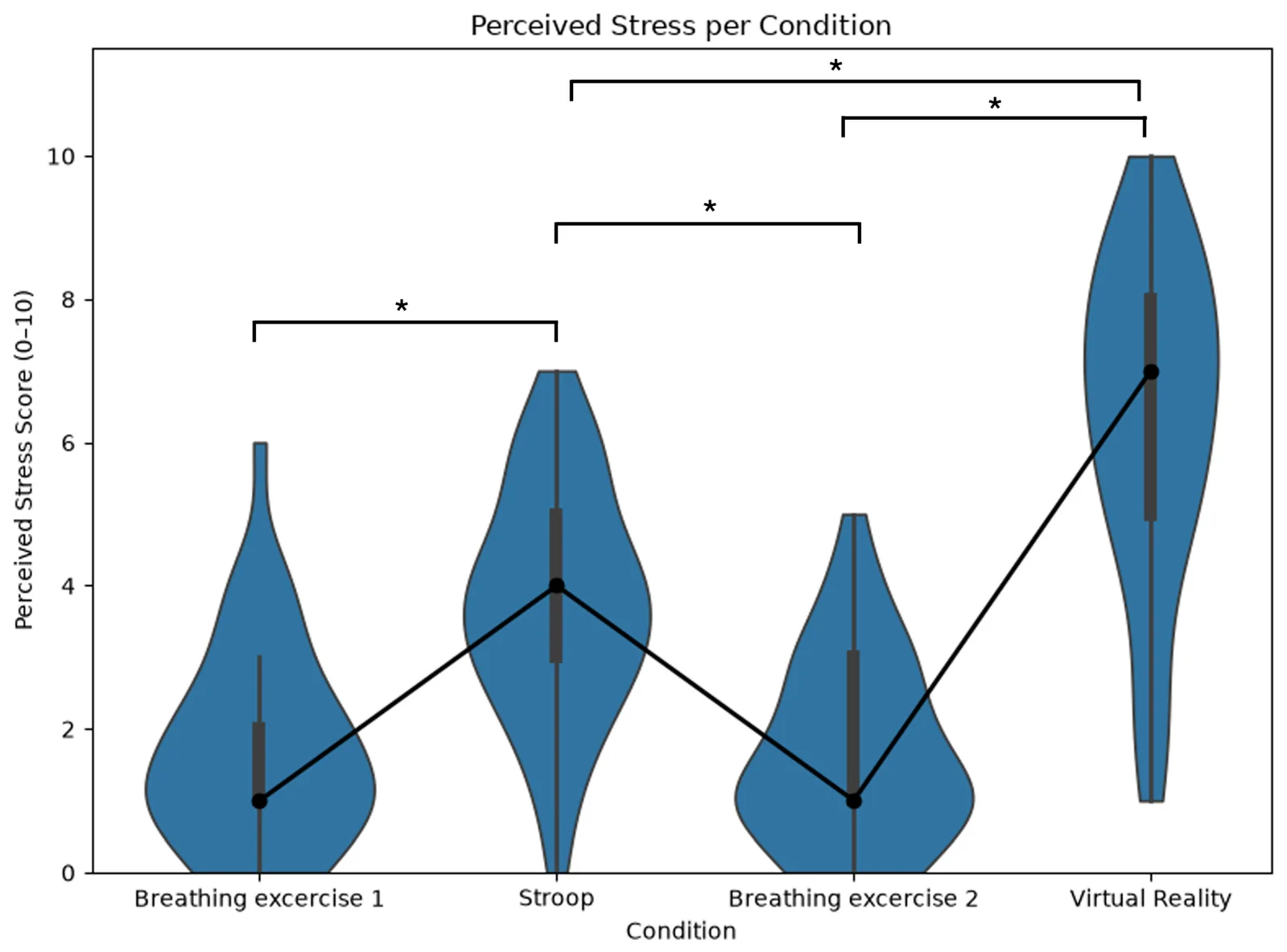
Perceived stress ratings differed between conditions. Violin plot showing the distribution of perceived stress scores (0–10) across four experimental conditions for the participants retained for physiological signal analysis (*n* = 41). The width of each violin reflects the distribution of responses, and dots connected by the black line indicate median scores per condition. Horizontal lines with * indicate statistically significant pairwise differences based on Bonferroni-corrected post hoc tests *p* < 0.0083).

Post hoc pairwise comparisons (see Figure 5) with Bonferroni correction indicated that the VR condition was perceived as significantly more stressful than the Stroop (*p* = 0.005) and Breathing Exercise conditions (*p* < 0.0001), and the Stroop condition was significantly more stressful than both breathing exercise 1 and breathing exercise 2 (*p* < 0.0001). No significant difference was found between the two breathing exercises (*p* = 1.000). All significant pairwise differences were associated with large effect sizes (r > 0.80). These results suggest a clear gradation in perceived stress, increasing from relaxation tasks to cognitive challenge during the Stroop task, and peaking in the immersive VR environment.

### 3.2. Characteristics EDA Signal

On average, the synchronized signals of the wearables had a total duration of 1963.3 s (SD ± 172.5 s). Overall, the average amplitude of the complete EDA signal measured by the Shimmer3 GSR+ device was 2.44 µS (SD ± 1.40), whereas the EmbracePlus on average measured 0.61 µS (SD ± 1.66). The corresponding median (Q1–Q3) values were 2.40 µS (1.08–3.72) and 0.21 µS (0.09–0.40), respectively. See Table 1 for all metrics per event. Boxplots of all EDA metrics on the original and LN(x + 1)-transformed scales are provided in Supplementary File S3.

### 3.3. Signal-Level Agreement

**Figure 6:**
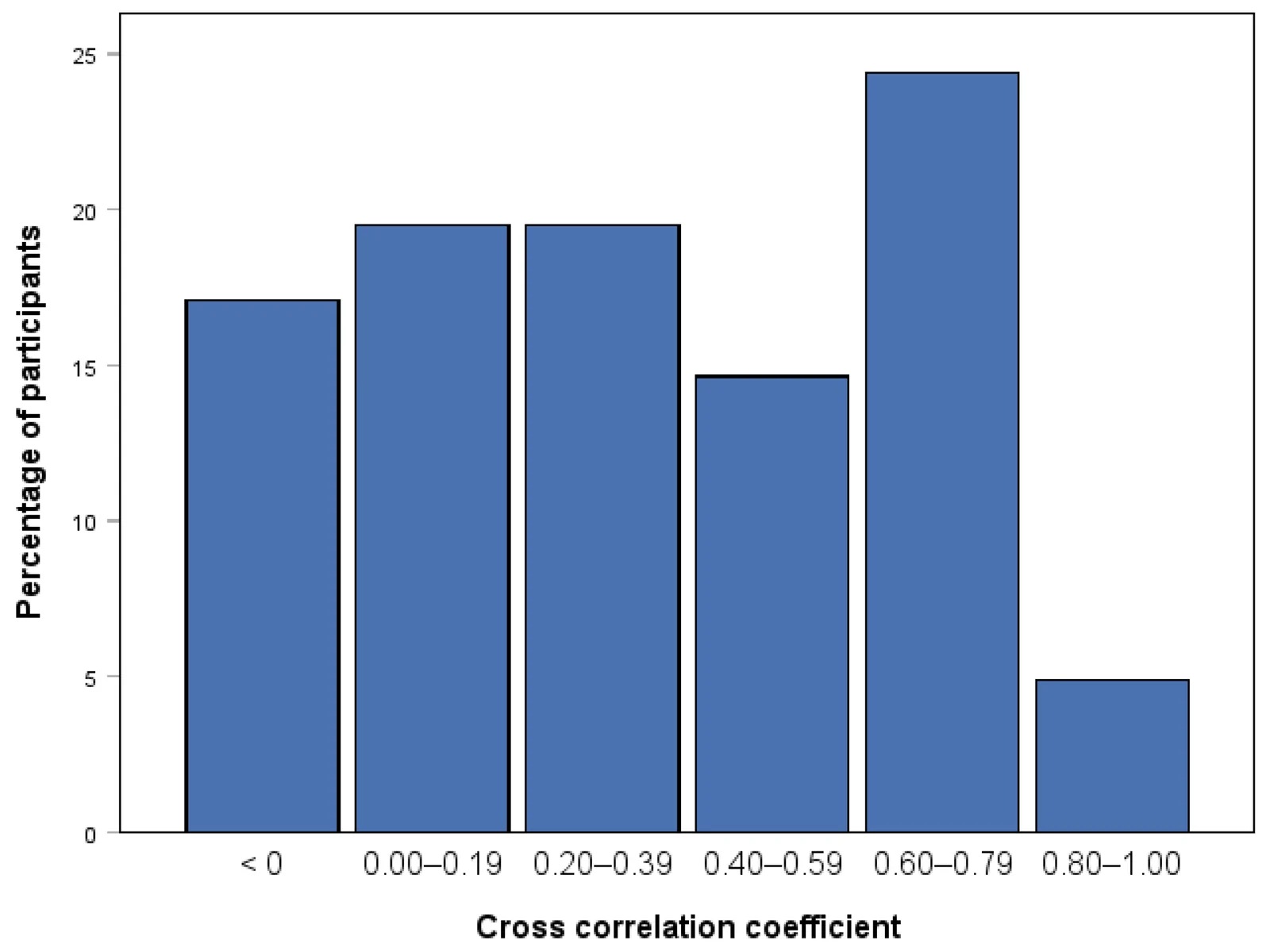
Histogram of the optimal cross-correlation. Cross-correlations were found for each participant between −4 and + 4 lags in data points (i.e., a 2 s timeframe).

**Figure 7:**
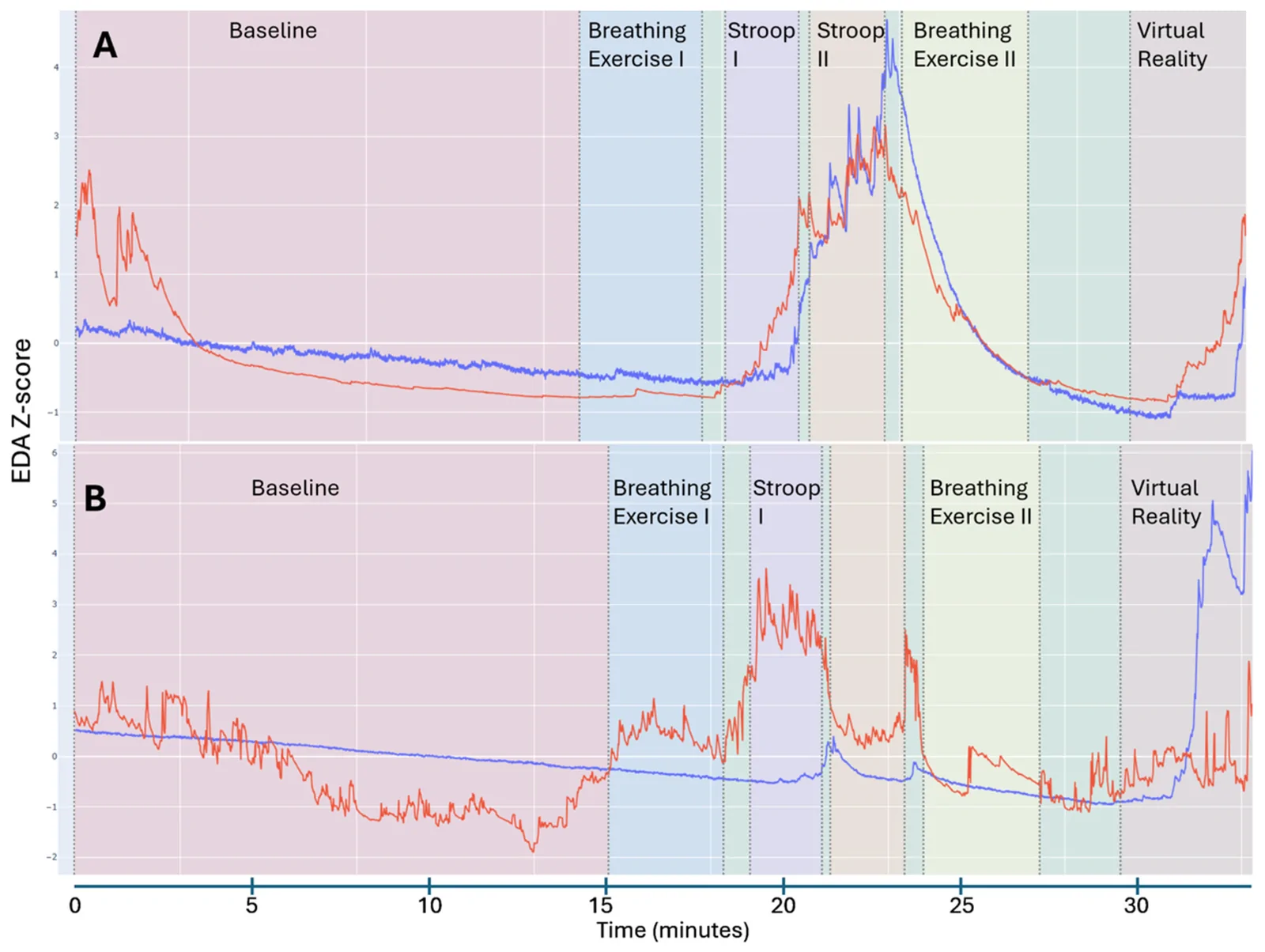
Examples of high and low signal-level agreement between devices. Normalized and detrended EDA signals are shown for the EmbracePlus (blue line) and Shimmer3 GSR+ (red line) across the full experimental protocol. Panel (**A**) shows a participant with high cross-correlation between devices (r = 0.84; subject 31), whereas Panel (**B**) shows a participant with low cross-correlation (r = 0.00; subject 14).

On average, the cross-correlation over the entire signal was 0.30, which could be considered weak-to-moderate [40]. Seven of 41 (17%) participants showed a negative correlation. In 12/41 (29%) of the participants, the correlation was >0.60 (see Figure 6). Figure 7 illustrates two signals with a high and low cross-correlation during the experiment.

Secondary analysis was performed, in which cross-correlations were calculated between the start of breathing exercise I and the end of breathing exercise II, as during this timeframe participants sat still and relatively few movement artifacts were expected. This also yielded a weak-to-moderate average cross-correlation of 0.35, with 13/41 (32%) of the participants having a cross-correlation> 0.60.

### 3.4. Parameter-Level Agreement

**Figure 8:**
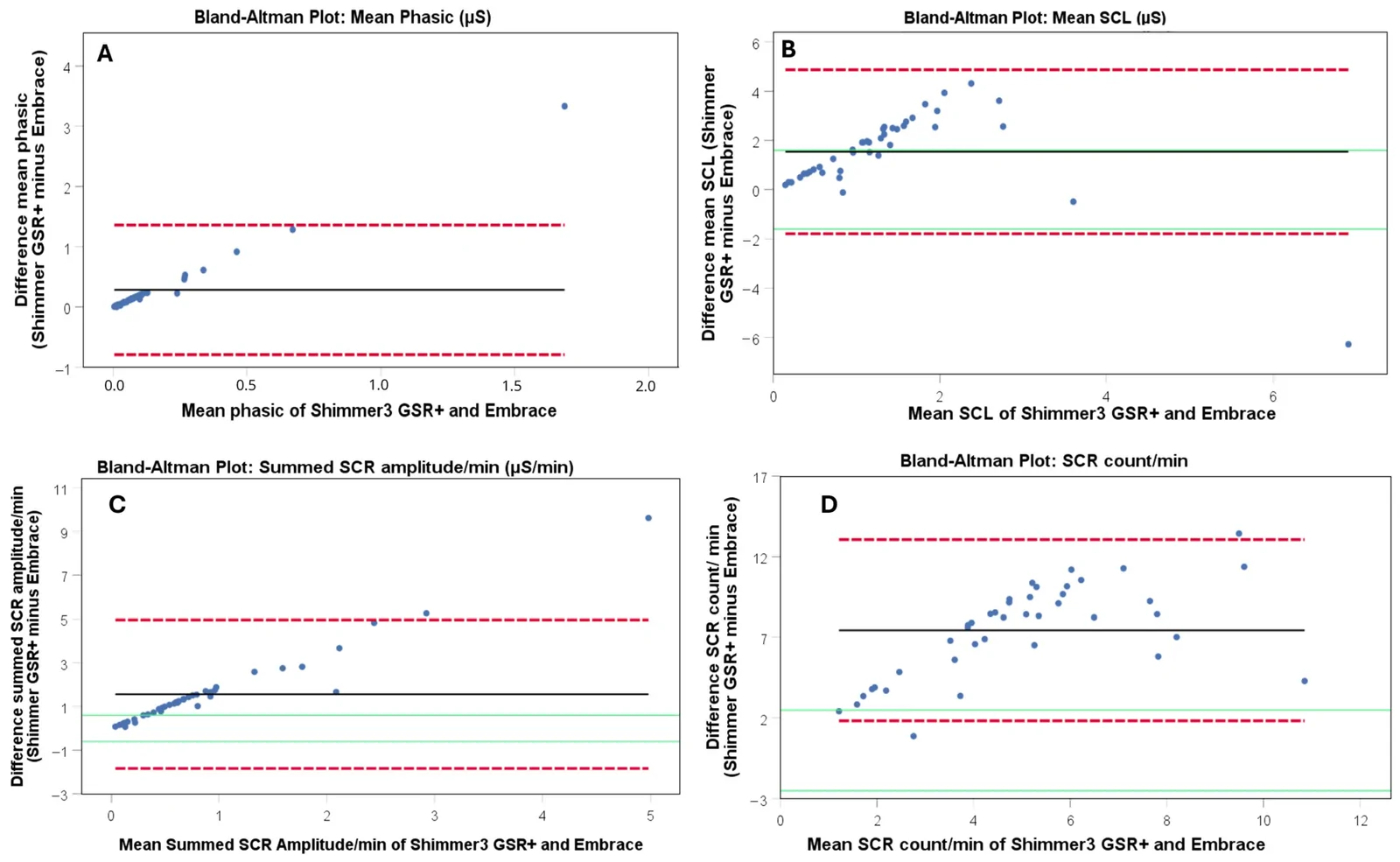
Bland–Altman plots of parameter-level agreement between devices. Bland–Altman plots are shown for (**A**) mean phasic (µS), (**B**) mean SCL (µS), (**C**) summed SCR amplitude/min (µS/min), and (**D**) SCR count/min. Differences were computed as Shimmer3 GSR+ minus EmbracePlus; the black solid line indicates mean bias, the red dashed lines indicate the 95% limits of agreement (bias ± 1.96 × SD), and the green lines indicate the a priori chosen acceptable boundaries. For the mean phasic activity, no a priori acceptable boundary was available.

Figure 8 presents Bland–Altman plots for mean phasic activity (µS), mean SCL (µS), Summed SCR amplitude/min (µS/min), and SCR count/min (SCRs/min), with differences defined as Shimmer3 GSR+ minus EmbracePlus. Across all four metrics, the mean difference (bias) was positive, indicating that Shimmer3 GSR+ yielded systematically higher values than EmbracePlus. Of the three metrics with an available a priori acceptable boundary, only a small proportion of observations lay inside those boundaries (SCL: 18/41 (44%), summed SCR amplitude/min: 10/41 (24%), SCR count/min: 2/41 (5%)), indicating that many participant-level differences exceeded the predefined acceptable error margins. This pattern persisted after LN(x + 1) transformation (SCL: 6/41 (15%), summed SCR amplitude/min: 4/41 (10%), SCR count/min: 1/41 (2%)), with most participant-level differences remaining outside the predefined acceptable margins (see Supplementary File S4).

Visual inspection further suggested that inter-device differences tended to increase with increasing measurement magnitude for several metrics, indicating possible proportional bias and/or changes in variability across levels. Isolated extreme observations were visible at the upper end of the range. Regression analyses with BCa 95% bootstrap confidence intervals indicated positive proportional bias for mean phasic activity, summed SCR amplitude/min and SCR count/min (see Table 2). For SCL, no evidence of proportional bias was found in the primary regression analysis, although the sensitivity analysis excluding the most influential observation indicated positive proportional bias.

Secondary analyses using LN(x + 1)-transformed device-specific values to address skewness and magnitude-dependent variability showed that the proportional-bias findings were partly scale-dependent (see Supplementary File S4). Positive proportional bias remained evident for mean phasic and summed SCR amplitude/min. It was no longer evident for SCL. For SCR count/min, the direction of the regression coefficient reversed, from positive proportional bias on the original scale to negative proportional bias on the transformed scale.

### 3.5. Event-Level Agreement

Within-device results for the Stroop and VR events are summarized in Table 3. For the Stroop stressor, Shimmer3 showed significant event effects for all four EDA metrics, with post hoc comparisons indicating increases from baseline 1 to Stroop 1 and/or Stroop 2. For EmbracePlus, event effects were metric-dependent: Mean phasic activity and mean SCL showed significant increases from breathing exercise 1 to Stroop 1 (mean phasic and SCL) and Stroop 2 (mean SCL), whereas summed SCR amplitude/min and SCR count/min did not. For the VR height stressor, both devices showed increased EDA metrics from breathing exercise 2 to VR, indicating that both wearables captured the VR-related increase in EDA. Event-level figures for mean phasic activity, mean SCL, summed SCR amplitude/min, and SCR count/min for both original data and log-transformed data are provided in Supplementary File S5 for the EmbracePlus and in Supplementary File S6 for the Shimmer3 GSR+.

**Figure 9:**
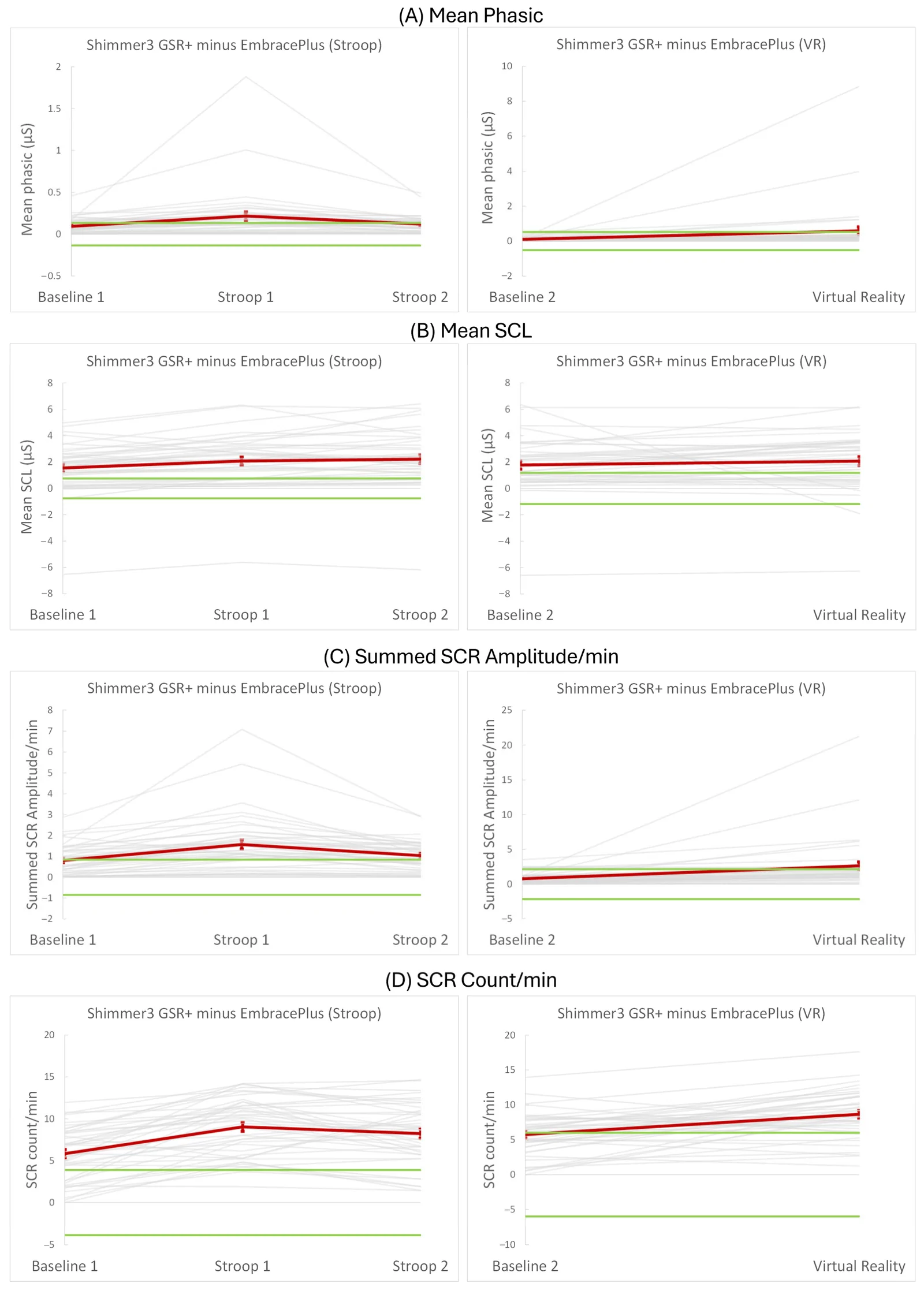
Inter-device difference plots across the Stroop and VR event sets. Inter-difference plots for (**A**) mean phasic (µS), (**B**) mean SCL (µS), (**C**) summed SCR amplitude/min (µS), and (**D**) SCR count/min, computed as Shimmer3 GSR+ minus EmbracePlus. For each metric, the left panel shows the Stroop event set (Breathing Exercise 1 as baseline 1, Stroop 1, Stroop 2) and the right panel shows the VR event set (Breathing Exercise 2 as baseline 2, Virtual Reality). Grey lines represent individual participants, and the red line indicates the group means ± SE. Green horizontal lines indicate the a priori acceptability boundaries (±|reference effect|), derived from the corresponding Shimmer3 baseline-to-stressor change. For the Stroop event set, the reference effect was defined as the change from Breathing Exercise 1 to Stroop 1; for the VR event set, it was defined as the change from Breathing Exercise 2 to Virtual Reality.

Regarding inter-device differences, difference plots (Shimmer3—EmbracePlus; Figure 9) were predominantly positive across metrics, indicating systematically higher values in Shimmer3. During the Stroop stress task (Breathing exercise 1, Stroop 1, Stroop 2), the mean difference remained consistently above zero and was frequently approached or exceeded the upper a priori acceptability boundary, suggesting that the between-device discrepancy was often comparable to, or larger than, the Stroop-related reference effect used to define acceptable error. During the VR stress task (Breathing exercise 2 to VR), mean differences increased from Breathing exercise 2 to VR for multiple outcomes; in several cases the mean difference was already close to the upper boundary at Breathing exercise 2 and exceeded it most clearly during VR, indicating larger between-device discrepancies in the higher-arousal context. Analyses using LN(x + 1)-transformed outcomes showed the same directional pattern and did not change the overall acceptability interpretation; however, expressing differences on the log scale (log-units) altered the apparent contrast between devices in most metrics. Because log-scale differences are not directly interpretable as absolute µS or SCR count/min differences, we report primary results on the original scale and include log-transformed visualizations in Supplementary File S7.

## 4. Discussion

The aim of this study was to evaluate the validity of the EmbracePlus for wrist-worn EDA measurement by comparing it with the Shimmer3 GSR+ across signal-, parameter-, and event-level analyses [13] in a standardized laboratory protocol. Overall, the findings indicate that the EmbracePlus and Shimmer3 GSR+ do not measure EDA interchangeably. Signal-level agreement was weak to moderate, and parameter-level analyses showed systematic positive inter-device bias, with Shimmer3 GSR+ consistently yielding higher values than EmbracePlus. The event-level difference plots also showed that inter-device differences were predominantly positive and frequently approached or exceeded the *a priori* acceptability boundaries, indicating that the disagreement between devices was often comparable to, or larger than, the reference baseline-to-stressor effect itself. When considering stressor-related EDA reactivity within each device, the Shimmer3 GSR+ showed significant increases across all EDA metrics during both the Stroop and VR tasks relative to baseline. For the EmbracePlus, the ability to capture stressor-related EDA changes depended on the stressor and the metric: during the stronger VR stressor, all EDA metrics increased significantly relative to baseline, whereas during the milder Stroop task only mean phasic and mean SCL showed clear increases, while summed SCR amplitude/min and SCR count/min did not. Taken together, these findings suggest that the practical validity of the EmbracePlus depends on the intended purpose, the selected EDA metric, and the intensity of the stressor being assessed.

A likely explanation for this pattern is that the EmbracePlus and Shimmer3 GSR+ differ in their recording characteristics. In the present study, both devices measured EDA on the ventral wrist at 4 Hz, with the Shimmer3 GSR+ using wet Ag/AgCl electrodes, and the EmbracePlus using dry electrodes. The Shimmer3 GSR+ uses an internal resistor network and selectable measurement ranges, including an auto-range function, whereas the EmbracePlus has a specified EDA measurement range of 0.01–100 µS. Differences in electrode technology, measurement range and architecture, as well as potential differences in calibration, gain, and skin–electrode contact impedance, may therefore contribute to the observed proportional bias. The present study was not designed to disentangle the contributions of these individual factors, including device characteristics and electrode configuration.

The matched wrist placement is also important when interpreting the present findings. By recording both devices at the wrist, differences attributable to anatomical measurement site were minimized, such that the comparison primarily reflects agreement between two wrist-based EDA measurement systems. However, this design does not establish equivalence of the EmbracePlus with conventional palmar EDA recordings. This distinction is relevant because palmar sites are conventionally used for psychophysiological EDA assessment, whereas wrist EDA may additionally be influenced by thermoregulatory sweating and may differ in its response characteristics from palmar EDA [6,7,11]. Future studies incorporating simultaneous wrist and palmar recordings could further determine how EmbracePlus measurements relate to conventional psychophysiological EDA assessment.

The wrist placement also affects interpretation of the a priori parameter-level acceptability boundaries. These were based on biologically plausible EDA ranges from the conventional literature, using palmar and volar finger sites as the standard recording locations. However, absolute SCL and SCR-related measures have been reported to be lower at the wrist than at palmar or finger sites [7,11]. Therefore, these boundaries may be relatively permissive for wrist recordings. Nevertheless, only 44% of SCL, 24% of summed SCR amplitude/min, and 5% of SCR count/min differences fell within these boundaries, so narrower wrist-specific criteria would not alter the conclusion of limited parameter-level agreement. In contrast, the event-level boundaries were based on the baseline-to-stressor effect measured by the wrist-worn Shimmer3 GSR+ itself, as proposed by Van Lier et al. [13] and were therefore not derived from palmar EDA ranges.

The ability of the Shimmer3 to capture EDA reactivity to both stressors across all EDA metrics is in line with previous studies using the Shimmer3 for EDA measurements on the fingers with dry electrodes [22,23,41,42,43]. In addition, the predecessor of the EmbracePlus, the Empatica E4, has previously been shown to capture EDA changes during stressful events using dry ventral wrist electrodes. At the same time, average EDA values reported for the Empatica E4, ranging from 1.4 (SD ± 2.0) to 2.2 (SD ± 2.7) µS [44], appear higher than the mean value of 0.61 µS (SD ± 1.66) observed for the EmbracePlus in the present study. Other recent studies also suggest that relatively low EDA values are recorded by the EmbracePlus [15,18,45]. This pattern is further supported by Acan et al. [19], who simultaneously recorded EDA with the EmbracePlus and Empatica E4 and found systematically lower absolute EDA amplitudes for the EmbracePlus, while correspondence was stronger for phasic than tonic EDA metrics. Although their study focused on synchronization and cross-device consistency during prolonged recordings rather than physiological reactivity during controlled stress induction, these findings support the interpretation that the lower absolute EDA values observed in the present study may partly reflect device-specific recording characteristics. In particular, the Empatica E4 used 8 mm dry wrist electrodes available in stainless steel SUS316L or silver (AG), whereas the EmbracePlus uses 6 mm ventral dry stainless steel 316L electrodes. Taken together, the present results support the interpretation that the EmbracePlus records lower EDA values than would be expected based on earlier wrist-worn devices and research-grade recordings.

In the present study, the lower values recorded by the EmbracePlus were visible in both the raw and processed EDA signal, such as mean SCL, mean phasic activity, SCR count/min, and summed SCR amplitude/min. The present findings further suggest that these lower values are not only relevant for absolute signal levels, but also for how sensitively stress-related changes can be captured and how the signal should be processed. This was reflected in the event-level results, where perceived stress was higher during the VR task than during the Stroop task. The EmbracePlus showed significant increases across all four EDA metrics during the more strongly perceived VR stressor across all four metrics, whereas during the milder Stroop task no effects were found for summed SCR amplitude/min or SCR count/min. Both of these metrics depend on a predefined amplitude threshold, meaning that only responses exceeding this threshold are classified as physiologically plausible SCRs. Conventionally, this threshold is set between 0.01 and 0.05 µS [6,7]. The present findings suggest that even the lowest conventional threshold of 0.01 µS may be less suitable for the EmbracePlus in its current wrist-worn dry-electrode configuration, as no Stroop-related differences were detected for these threshold-based SCR metrics. In contrast, stress-related differences during the Stroop task were detected when using mean phasic and mean SCL, which do not rely on such thresholds. Similar concerns have been reported for wearable EDA processing. Van Lier et al. [13] explored a substantially lower SCR threshold of 0.001 µS for the Empatica E4, although this did not improve agreement with their reference device. Other work has proposed normalization of the phasic signal before peak detection when applying SCR detection across signals with different amplitude ranges [46]. Recent exploratory work further illustrates the sensitivity of SCR outcomes to the processing approach: despite lower tonic and overall EDA values, the EmbracePlus yielded more detected SCRs than the Empatica E4 [18]. However, these devices were assessed in separate participant cohorts and using a different preprocessing and peak-detection pipeline, limiting direct comparison with the present findings. Together, these results suggest that SCR-based outcomes may be particularly dependent on both device characteristics and processing choice, and that device- or signal-specific processing may be preferable to direct application of conventional absolute thresholds.

These findings indicate that the performance of the EmbracePlus is context-dependent and metric-dependent, which is in line with the rationale of van Lier et al. [13], who emphasize that validity should be evaluated in relation to the intended context of use. From a practical perspective, the present findings suggest that the EmbracePlus may be more suitable for detecting greater stress-related changes and for monitoring within-subject trends over time than for obtaining precise absolute EDA values. In particular, the present results indicate that baseline-relative interpretation may be more informative than interpretation of raw absolute values when using this device. This is relevant because the systematic inter-device bias and the frequent exceedance of the a priori acceptability boundaries indicate that absolute agreement with a research-grade comparator was limited. At the same time, the event-level findings show that the device can still capture greater stress-related changes, especially when using metrics that are less dependent on predefined SCR thresholds, such as mean phasic activity and mean SCL. In addition, future work could explore whether less amplitude-dependent approaches, including frequency- or time-frequency-based analyses [47], provide additional value for analyzing lower-amplitude EDA signals.

The wrist-worn configuration may facilitate prolonged ambulatory use, as dry-electrode wrist EDA has been identified as a promising approach for prolonged daily life monitoring [11], and recent studies using the EmbracePlus indicate good practical wearability, with most participants reporting little or no interference during daily activities and successful use over multi-week monitoring periods [18,48]. At the same time, ambulatory EDA recordings remain susceptible to artifacts such as motion [49]. These considerations are important when translating the present laboratory findings to prolonged daily life monitoring.

A methodological strength of the present study is that validity was evaluated across multiple levels of analysis, following the framework of van Lier et al. [13]. This made it possible to distinguish between raw signal similarity, agreement in derived parameters, and the ability to detect stress-related changes at the event level, thereby providing a more nuanced evaluation than a single overall validity estimate would allow. In addition, both devices were worn simultaneously on the same wrist, which reduced between-subject and contextual variation in the comparison. The study design also included a 15 min wear-in period before the active tasks. This was intended to allow skin contact and signal quality to stabilize. Furthermore, the interpretation of the event-level findings was strengthened by the inclusion of perceived stress ratings, which confirmed that the VR task was experienced as more stressful than the Stroop task. Finally, by examining multiple tonic and phasic EDA metrics, the study was able to show that validity depended on the selected outcome measure and the associated signal-processing approach.

Several limitations should be considered when interpreting these findings. The signal-level agreement showed substantial between-participant variability, as reflected in the range of cross-correlation coefficients. As the present study was not designed to determine which participant- or recording-related factors explained this variability, future research is needed to examine whether it is influenced by factors such as skin characteristics, contact quality, movement, or other person-specific recording conditions. A further limitation concerns sample size. Although 53 participants were recruited, 41 remained for the paired analyses after signal-quality exclusions, which is below the approximately 55 participants recommended by Van Lier et al. [13] for their complete validation protocol. Importantly, this recommendation is based on different assumptions across the three validation levels rather than on a single EDA-specific sample-size calculation, and these calculations are not directly transferable to the present design. Nevertheless, the reduced sample size may have limited the precision of the agreement estimates and the ability to detect smaller effects, and replication in larger samples is therefore warranted. Another methodological limitation concerns skin preparation with 70% isopropyl alcohol before sensor placement. Routine pretreatment of active EDA recording sites is generally discouraged [6]. While a 15 min wear-in period preceded the active tasks, it is unclear whether this was sufficient for electrode–skin stabilization, particularly for the dry EmbracePlus electrodes, and whether alcohol affected the dry and pre-gelled electrodes differently. Future studies should avoid routine alcohol-based skin preparation [50] and determine the stabilization time required for the EmbracePlus dry-electrode configuration.

The study was conducted in a standardized laboratory setting with two specific acute stressor paradigms in a fixed order. Although this order was chosen to reduce potential carryover from the VR condition, which elicited higher perceived stress, to the Stroop task, differences between conditions cannot be fully separated from possible order, carryover, habituation, or fatigue effects. Moreover, the present results do not show whether similar patterns would also be observed during ambulatory monitoring, during other types of stressors, or in clinical populations. Furthermore, the breathing exercises were used as low-arousal reference conditions, although deep breathing can itself evoke respiration-related EDA fluctuations that resemble SCRs [6]. These responses may diminish with repeated inspirations due to habituation [51]. Given the slow 4–7–8 breathing pattern used in the present study, respiration-related SCRs may therefore have occurred intermittently, but their contribution could not be quantified because respiration was not recorded. This also affects interpretation of the event-level agreement, as respiration-related elevations in EDA during the breathing conditions could reduce the estimated baseline-to-stressor reference effect and thereby narrow the corresponding acceptability boundaries, as defined in the framework of Van Lier et al. [13].

Several analytical considerations should also be noted. Because the parameter-level metrics were aggregated across the retained recording, condition-specific over- and underestimation may partly offset each other. The complementary event-level analyses were therefore important for evaluating device performance across the experimental conditions. Although the findings suggest that conventional threshold-based SCR processing may be less suitable for the EmbracePlus in its current wrist-worn dry-electrode configuration, systematically determining an alternative optimal threshold or alternative processing strategy for this device fell outside the scope of the present study.

Finally, the comparison was restricted to one specific research-grade comparator and one specific placement configuration at the wrist. The extent to which these findings generalize to other wrist-worn EDA devices, preprocessing pipelines, or electrode configurations remains to be established in future research.

## 5. Conclusions

In conclusion, the present study shows that the EmbracePlus records systematically lower wrist-worn EDA values than the Shimmer3 GSR+, with limited agreement across the signal-, parameter-, and event-level analyses. Although the EmbracePlus was able to detect within-subject baseline-to-stressor EDA changes, inter-device discrepancies at the event level were still often large relative to the reference effect. The EmbracePlus therefore appears more suitable for detecting greater within-subject stress-related changes than for interpreting precise absolute EDA values, particularly when conventional threshold-based SCR metrics are used. Accordingly, lower-amplitude dry-electrode EDA signals may not be optimally processed using absolute SCR thresholds between 0.01 and 0.05 µS, as this may reduce sensitivity to milder stress-related changes. Future research is needed to determine which preprocessing and metric choices are most appropriate for devices such as the EmbracePlus, and whether these findings generalize beyond this specific wrist-worn laboratory setup.

## Figures and Tables

**Table 1:** Descriptive values of EDA metrics. For each event, four metrics were analyzed for the EmbracePlus and Shimmer3 GSR+. Values are reported as median (Q1–Q3).

EmbracePlus	Mean Phasic (µS)	Mean SCL (µS)	Summed SCR Amplitude/min (µS/min)	SCR Count/min
Breathing exercise 1	0.00 (0.00–0.00)	0.11 (0.08–0.30)	0.00 (0.00–0.01)	0.00 (0.00–0.56)
Stroop 1	0.00 (0.00–0.00)	0.14 (0.09–0.31)	0.00 (0.00–0.04)	0.00 (0.00–1.84)
Stroop 2	0.00 (0.00–0.00)	0.22 (0.11–0.40)	0.00 (0.00–0.03)	0.00 (0.00–1.88)
Breathing exercise 2	0.00 (0.00–0.00)	0.21 (0.10–0.36)	0.00 (0.00–0.01)	0.00 (0.00–0.30)
Virtual Reality	0.01 (0.00–0.03)	0.31 (0.13–0.83)	0.06 (0.01–0.35)	1.90 (0.40–5.57)
Shimmer3 GSR+	Mean phasic (µS)	Mean SCL (µS)	Summed SCR amplitude/min (µS/min)	SCR count/min
Breathing exercise 1	0.07 (0.02–0.15)	1.99 (1.09–3.01)	0.73 (0.22–1.29)	7.02 (4.41–8.83)
Stroop 1	0.14 (0.08–0.24)	2.54 (1.40–3.69)	1.34 (0.82–2.11)	10.84 (7.57–13.25)
Stroop 2	0.11 (0.04–0.18)	2.38 (1.57–4.24)	1.08 (0.44–1.64)	9.47 (6.68–12.68)
Breathing exercise 2	0.05 (0.02–0.15)	2.36 (1.09–3.39)	0.46 (0.21–1.18)	6.64 (4.56–8.33)
Virtual Reality	0.22 (0.16–0.36)	3.22 (1.50–3.99)	1.76 (1.30–2.75)	12.11 (10.59–14.20)

**Table 2:** Proportional bias.

EDA Metric	Analysis	*n*	B	BCa 95% CI
Mean phasic (µS)	Primary analysis	41	1.892	**[1.669; 1.941]**
	Sensitivity analysis	40	1.801	**[1.488; 1.931]**
Mean SCL (µS)	Primary analysis	41	−0.446	[−1.096; 1.768]
	Sensitivity analysis	40	0.888	**[0.087; 1.859]**
summed SCR amplitude/min (µS/min)	Primary analysis	41	1.802	**[1.432; 1.918]**
	Sensitivity analysis	40	1.649	**[1.196; 1.897]**
SCR count/min	Primary analysis	41	0.777	**[0.261; 1.325]**
	Sensitivity analysis	40	1.044	**[0.726; 1.396]**

Proportional bias was assessed using linear regression, with the inter-device difference (Shimmer3 GSR+ − EmbracePlus) as the dependent variable and the mean of both devices as the independent variable. Primary analyses included all valid participants (*n* = 41). Where the maximum Cook’s distance exceeded 1, a sensitivity analysis was performed after exclusion of the single most influential observation. B represents the unstandardized regression coefficient estimated from the original sample; a positive B indicates that the inter-device difference increases with measurement magnitude, whereas a negative B indicates that the difference decreases with measurement magnitude. Bias-corrected and accelerated (BCa) 95% confidence intervals excluding zero are shown in bold.

**Table 3:** Summary of event-level within-device analyses for EmbracePlus and Shimmer3 GSR+ across EDA metrics and event sets.

Device	EDA Metric	Event Set	*p*-Value	Effect Size	Significant Comparisons
EmbracePlus	Mean phasic (µS)	Stroop	***p* = 0.001**	W = 0.17	B1 < S1
		VR	***p* < 0.001**	r = 0.78	B2 < VR
	Mean SCL (µS)	Stroop	***p* < 0.001**	W = 0.75	B1 < S1 < S2, B1 < S2
		VR	***p* < 0.001**	r = 0.62	B2 < VR
	summed SCR amplitude/min (µS/min)	Stroop	*p* = 0.070	-	-
		VR	***p* < 0.001**	r = 0.74	B2 < VR
	SCR count/min	Stroop	*p* = 0.070	-	-
		VR	***p* < 0.001**	r = 0.77	B2 < VR
Shimmer3 GSR+	Mean phasic (µS)	Stroop	***p* < 0.001**	W = 0.47	B1 < S1 > S2
		VR	***p* < 0.001**	r = 0.84	B2 < VR
	Mean SCL (µS)	Stroop	***p* < 0.001**	W = 0.38	B1 < S1, B1 < S2
		VR	***p* < 0.001**	r = 0.58	B2 < VR
	summed SCR amplitude/min (µS/min)	Stroop	***p* < 0.001**	W = 0.51	B1 < S1 > S2, B1 < S2
		VR	***p* < 0.001**	r = 0.86	B2 < VR
	SCR count/min	Stroop	***p* < 0.001**	W = 0.52	B1 < S1, B1 < S2
		VR	***p* < 0.001**	r = 0.87	B2 < VR

For the Stroop set (Breathing exercise 1, Stroop 1, Stroop 2), differences between conditions were tested using Friedman tests; for the VR set (Breathing exercise 2, VR), differences were tested using Wilcoxon signed-rank tests. Bold *p*-values indicate statistically significant results (*p* < 0.05). Effect sizes are reported as Kendall’s W for Friedman tests and as r for Wilcoxon tests. Significant comparisons are shown only when significant (family-wise error rate = 0.05) and were Bonferroni-corrected where relevant. B1 = Breathing exercise 1, B2 = Breathing exercise 2, S1 = Stroop 1, S2 = Stroop 2, VR = Virtual Reality.

## Data Availability

The data underlying the findings of this study cannot be made publicly available because the informed consent obtained from participants did not include permission for unrestricted public sharing of their data. In accordance with the consent provided by the participants, the data may only be shared for research conducted by Fontys University of Applied Sciences and its research partners. The dataset is archived in DataverseNL under DOI: doi.org/10.34894/2HBZVS, with access to the data restricted. Researchers may submit a data access request to Research Data Management, Fontys University of Applied Sciences, rdm@fontys.nl. If a request is approved, an appropriate research partnership with Fontys University of Applied Sciences must be established before access to the data is provided, in accordance with the conditions of the informed consent and applicable institutional procedures.

## Supplementary Materials

The following supporting information can be downloaded at: https://www.mdpi.com/article/10.3390/s26216795/s1. Supplementary File S1: Python code used for EDA preprocessing. Supplementary File S2: Distributions of inter-device differences at the parameter level. Supplementary File S3: Boxplots of EDA metrics per event and device. Supplementary File S4: LN(x + 1)-scaled Bland–Altman plots. Supplementary File S5: Event-level within-device plots for the EmbracePlus. Supplementary File S6: Event-level within-device plots for the Shimmer3 GSR+. Supplementary File S7: LN(x + 1) scaled inter-device difference plots.

## References

[1] Dhabhar F.S. (2018). The short-term stress response—Mother nature’s mechanism for enhancing protection and performance under conditions of threat, challenge, and opportunity. Front. Neuroendocr..

[2] Epel E.S., Crosswell A.D., Mayer S.E., Prather A.A., Slavich G.M., Puterman E., Mendes W.B. (2018). More than a feeling: A unified view of stress measurement for population science. Front. Neuroendocr..

[3] Snippe E., Myroniuk S., Meyer M., Bültmann U., Scheibe S., Jeronimus B.F. (2026). The ambiguous conceptualization of stress: Stressor, stress response, and health outcomes. PsyArXiv Prepr..

[4] Selye H. (2013). Stress in Health and Disease.

[5] Schmidt P., Reiss A., Dürichen R., Laerhoven K.V. (2019). Wearable-Based Affect Recognition-A Review. Sensors.

[6] Boucsein W. (2012). Electrodermal Activity.

[7] Dawson M.E., Schell A.M., Filion D.L. (2007). The electrodermal system. Handbook of Psychophysiology.

[8] Georgiev G.V., Nagai Y., Noda S., Junaidy D.W., Taura T. An investigation of vehicle interface operation comfort. Proceedings of the DS 75-7: Proceedings of the 19th International Conference on Engineering Design (ICED13), Design for Harmonies.

[9] Klimek A., Mannheim I., Schouten G., Wouters E.J.M., Peeters M.W.H. (2023). Wearables measuring electrodermal activity to assess perceived stress in care: A scoping review. Acta Neuropsychiatr..

[10] Tamantini C., Laura Cristofanelli M., Fracasso F., Umbrico A., Cortellessa G., Orlandini A., Cordella F. (2025). Physiological Sensor Technologies in Workload Estimation: A Review. IEEE Sens. J..

[11] van der Mee D.J., Gevonden M.J., Westerink J., de Geus E.J.C. (2021). Validity of electrodermal activity-based measures of sympathetic nervous system activity from a wrist-worn device. Int. J. Psychophysiol..

[12] Schoenmakers M., Saygin M., Sikora M., Vaessen T., Noordzij M., de Geus E. (2025). Stress in action wearables database: A database of noninvasive wearable monitors with systematic technical, reliability, validity, and usability information. Behav. Res. Methods.

[13] van Lier H.G., Pieterse M.E., Garde A., Postel M.G., de Haan H.A., Vollenbroek-Hutten M.M.R., Schraagen J.M., Noordzij M.L. (2020). A standardized validity assessment protocol for physiological signals from wearable technology: Methodological underpinnings and an application to the E4 biosensor. Behav. Res. Methods.

[14] Badawi A., Elmoghazy S., Choudhury S., Elgazzar S., Elgazzar K., Burhan A.M. (2025). Multimodal Detection of Agitation in People with Dementia in Clinical Settings: Observational Pilot Study. JMIR Aging.

[15] Van Swieten M., De Looff P., Vandernagel J., Bouwmeester S., Didden R. (2025). Listening to music is associated with reduced physiological and subjective stress in people with mild intellectual disabilities: A biofeedback study. Res. Dev. Disabil..

[16] Sinichi M., Gevonden M.J., Krabbendam L. (2025). Quality in Question: Assessing the Accuracy of Four Heart Rate Wearables and the Implications for Psychophysiological Research. Psychophysiology.

[17] Žagar D., Geršak G. (2025). Performance Comparison of Wired and Wireless Electrodermal Activity Sensors During a Simulated Port Approach Maneuver. Electronics.

[18] Brandebusemeyer C., Georgi F., Arnrich B. (2026). Reliability Assessment of Wearable Technologies for Physiological Measurements: An Evaluation of Shimmer3 GSR+, Empatica E4, EmbracePlus, and Pixel Watch 2 Across Cognitive, Affective and Physical Activity Tasks. Sensors.

[19] Acan S., Valenzuela-Pascual C., Corponi F., Li B.M., Hidalgo-Mazzei D., Thijssen D., Vos G., Bogaerts S., Hermans E., Baratchi M. (2026). Standardized synchronization and validation pipeline for physiological biomarkers across multiple devices. Behav. Res. Methods.

[20] Houghton R., Martinetti A., Majumdar A. (2024). A Framework for Selecting and Assessing Wearable Sensors Deployed in Safety Critical Scenarios. Sensors.

[21] Sikora M., Zhao X., Van ’T Klooster J.-W., Koyuncu Z., De Geus E., Noordzij M. (2026). A systematic scoping review identifying effective virtual reality stress tasks inducing physiological reactivity for future wearables validation. Behav. Res. Methods.

[22] Giorgi A., Ronca V., Vozzi A., Sciaraffa N., di Florio A., Tamborra L., Simonetti I., Aricò P., Di Flumeri G., Rossi D. (2021). Wearable Technologies for Mental Workload, Stress, and Emotional State Assessment during Working-Like Tasks: A Comparison with Laboratory Technologies. Sensors.

[23] Ronca V., Martinez-Levy A.C., Vozzi A., Giorgi A., Aricò P., Capotorto R., Borghini G., Babiloni F., Di Flumeri G. (2023). Wearable Technologies for Electrodermal and Cardiac Activity Measurements: A Comparison between Fitbit Sense, Empatica E4 and Shimmer GSR3+. Sensors.

[24] Mavros P., J Wälti M., Nazemi M., Ong C.H., Hölscher C. (2022). A mobile EEG study on the psychophysiological effects of walking and crowding in indoor and outdoor urban environments. Sci. Rep..

[25] Sanchez-Comas A., Synnes K., Molina-Estren D., Troncoso-Palacio A., Comas-González Z. (2021). Correlation Analysis of Different Measurement Places of Galvanic Skin Response in Test Groups Facing Pleasant and Unpleasant Stimuli. Sensors.

[26] Nixon J.L., Cartmill B., Turner J., Pigott A.E., Brown E., Wall L.R., Ward E.C., Porceddu S.V. (2018). Exploring the prevalence and experience of mask anxiety for the person with head and neck cancer undergoing radiotherapy. J. Med. Radiat. Sci..

[27] Baghestani F., Kong Y., D’Angelo W., Chon K.H. (2024). Analysis of sympathetic responses to cognitive stress and pain through skin sympathetic nerve activity and electrodermal activity. Comput. Biol. Med..

[28] Zekioğlu A., Tok S., İşbilir M., Yılmaz S.E., Binboğa E., Dal N. (2026). Stress Responses and Recovery in Student Athletes: Heart Rate Variability and Skin Conductance Patterns Across Cognitive Challenges—A Pilot Study. Behav. Sci..

[29] Gromer D., Reinke M., Christner I., Pauli P. (2019). Causal Interactive Links Between Presence and Fear in Virtual Reality Height Exposure. Front. Psychol..

[30] Aktaş G.K., İlgin V.E. (2023). The Effect of Deep Breathing Exercise and 4–7-8 Breathing Techniques Applied to Patients After Bariatric Surgery on Anxiety and Quality of Life. Obes. Surg..

[31] Ghosh R., Deb N., Sengupta K., Phukan A., Choudhury N., Kashyap S., Phadikar S., Saha R., Das P., Sinha N. (2022). SAM 40: Dataset of 40 subject EEG recordings to monitor the induced-stress while performing Stroop color-word test, arithmetic task, and mirror image recognition task. Data Brief.

[32] Fiel Peres F. (2026). Effect sizes for nonparametric tests. Biochem. Medica.

[33] Empatica How to Access Avro Files with Python. https://support.empatica.com/hc/en-us/articles/17405877853981-How-to-access-Avro-files-with-Python.

[34] Makowski D., Pham T., Lau Z.J., Brammer J.C., Lespinasse F., Pham H., Schölzel C., Chen S.H.A. (2021). NeuroKit2: A Python toolbox for neurophysiological signal processing. Behav. Res. Methods.

[35] Greco A., Valenza G., Lanata A., Scilingo E., Citi L. (2016). cvxEDA: A Convex Optimization Approach to Electrodermal Activity Processing. IEEE Trans. Biomed. Eng..

[36] Greco A., Valenza G., Lázaro J., Garzón-Rey J.M., Aguiló J., De La Cámara C., Bailón R., Scilingo E.P. (2023). Acute Stress State Classification Based on Electrodermal Activity Modeling. IEEE Trans. Affect. Comput..

[37] Bland J.M., Altman D.G. (1999). Measuring agreement in method comparison studies. Stat. Methods Med. Res..

[38] Field A. (2018). Discovering Statistics Using IBM SPSS Statistics.

[39] Wood M. (2004). Statistical Inference using Bootstrap Confidence Intervals. Significance.

[40] Akoglu H. (2018). User’s guide to correlation coefficients. Turk. J. Emerg. Med..

[41] Wulvik A.S., Dybvik H., Steinert M. (2020). Investigating the relationship between mental state (workload and affect) and physiology in a control room setting (ship bridge simulator). Cogn. Technol. Work.

[42] Williams D., Hodge V.J., Wu C.Y. (2020). On the use of AI for Generation of Functional Music to Improve Mental Health. Front. Artif. Intell..

[43] Sciaraffa N., Di Flumeri G., Germano D., Giorgi A., Di Florio A., Borghini G., Vozzi A., Ronca V., Varga R., van Gasteren M. (2022). Validation of a Light EEG-Based Measure for Real-Time Stress Monitoring during Realistic Driving. Brain Sci..

[44] Vos G., Trinh K., Sarnyai Z., Rahimi Azghadi M. (2023). Ensemble machine learning model trained on a new synthesized dataset generalizes well for stress prediction using wearable devices. J. Biomed. Inform..

[45] Tomalin L.E., Kummer B.R., Campbell M.C., Erekat A.N., Cohen F., Robinson-Papp J., Mueller B.R. (2025). Can smartwatches predict migraines?Using machine learning (ML) with wearable-derived nocturnal autonomic nervous system (ANS) and sleep metrics for headache prediction. Res. Sq..

[46] Meijer A.L., Arts L.P.A., Gomez R., Van Den Broek E.L. (2023). Electrodermal activity: A continuous monitor of well-being. J. Smart Cities Soc..

[47] Lew R., Dyre B.P., Werner S., Wotring B., Tran T. (2008). Exploring the Potential of Short-Time Fourier Transforms for Analyzing Skin Conductance and Pupillometry in Real-Time Applications. Proc. Hum. Factors Ergon. Soc. Annu. Meet..

[48] Tomalin L.E., Kummer B.R., Campbell M.C., Erekat A., Wandner L., Cohen F., Clauw D., Robinson-Papp J., Mueller B.R. (2026). Wearable Nocturnal Autonomic and Sleep Biomarkers for Predicting Next-Day Headache and Identifying Nociplastic Pain in Patients with Migraine. J. Clin. Med..

[49] Böttcher S., Vieluf S., Bruno E., Joseph B., Epitashvili N., Biondi A., Zabler N., Glasstetter M., Dümpelmann M., Van Laerhoven K. (2022). Data quality evaluation in wearable monitoring. Sci. Rep..

[50] Boucsein W., Fowles D.C., Grimnes S., Ben-Shakhar G., Roth W.T., Dawson M.E., Filion D.L. (2012). Publication recommendations for electrodermal measurements. Psychophysiology.

[51] Seto-Poon M., Madronio M., Kirkness J.P., Amis T.C., Byth K., Lim C.L. (2005). Decrement of the skin conductance response to repeated volitional inspiration. Clin. Neurophysiol..

