# Supplementary material for: Validity Assessment of Wrist-Worn Electrodermal Activity Measurement During Relaxation and Acute Stressor Tasks: Comparing the EmbracePlus and Shimmer3 GSR+
Source: Sensors (Basel). 2026 Oct 27;26(21):6795. doi: 10.3390/s26216795 (PMC13765827; doi:10.3390/s26216795)
Supplement: Supplementary file 1 [file sensors-26-06795-s001.zip › Supplementary File S1 code.pdf]

Supplementary File S1: Python code used for EDA preprocessing

The raw EDA signal was first converted to z-scores and screened for short jump–return patterns indicative of artefacts. Detection was initialized using the first 5 s of the signal, after which the local baseline was defined as the mean of the last four samples retained in the reference buffer. A candidate artefact was identified when the signal deviated from this baseline by at least 0.75 z and returned in the opposite direction by at least 0.675 z within 1.5 s. All samples between the initial jump and the detected return were marked as artefact, and the marked interval was extended by 1 s on both sides to include the immediately surrounding, potentially unreliable samples. Artefact regions were then corrected in the original µS signal using linear interpolation between the last clean sample before the artefact block and the first clean sample after it. When only one clean anchor was available (at the border of the signal), the entire artefact block was replaced by that anchor value. The resulting signal is provided in the ‘Corrected EDA column’.

<u>Used python code:</u>

```
import numpy as np
from scipy.stats import zscore


def detect_eda_artifacts(
    raw_vals,
    sampling_rate,             # required: user-provided sampling rate (e.g., 4.0 Hz)
    thr_jump=0.75,             # z-score jump relative to baseline
    thr_return=0.675,          # z-score return relative to jump point (opposite direction)
    window_size=4,             # number of non-artifact points used for baseline
    return_window_sec=1.5      # maximum time for return in seconds
):
    """
    Detect short artifact spikes in an EDA signal based on z-scores.

    Artifact definition:
      - jump >= thr_jump z from the reference
        (mean of the last 'window_size' non-artifact points)
      - within 'return_window_sec', an opposite movement >= thr_return z
        relative to the jump point
      - all points from jump to return are marked as artifact

    Parameters
    ----------
    raw_vals : array-like
        Raw EDA values (µS).
    sampling_rate : float or int
        Sampling rate in Hz.
    thr_jump : float
        Minimum z-score jump for a candidate artifact.
    thr_return : float
        Minimum z-score return relative to the jump point within the return window.
    window_size : int
        Number of previous non-artifact points used for the baseline.
    return_window_sec : float
        Length of the return window in seconds.

    Returns
    -------
    artifact_mask : np.ndarray (bool)
        True where an artifact is present; False elsewhere.
    artifact_events : list[tuple[int,int]]
        List of artifact ranges as (start_idx, end_idx).
    """
    # --- sanity checks and derived constants ---
    if sampling_rate is None:
        raise ValueError("sampling_rate is vereist (geen default).")
    sr = float(sampling_rate)
    if sr <= 0:
        raise ValueError(f"sampling_rate moet > 0 zijn, got {sampling_rate!r}")

    return_window_samples = int(max(1, round(return_window_sec * sr)))
    init_ref_len = int(max(window_size, round(5 * sr)))  # 5 s initial reference
```

```
    # extend each artifact by ±1 second as an unreliable interval
    pad_sec = 1.0
    pad_samples = int(round(pad_sec * sr))  # may be 0 at very low sampling rates

    # --- convert data to z-scores ---
    raw_vals = np.asarray(raw_vals, dtype=float)
    if raw_vals.size == 0:
        return np.zeros(0, dtype=bool), []
    z_vals = zscore(raw_vals)

    n = len(z_vals)
    artifact_mask = np.zeros(n, dtype=bool)
    artifact_events = []

    # --- initialize reference buffer ---
    ref_init_end = min(init_ref_len, n)
    reference_buffer = list(z_vals[:ref_init_end])

    i = ref_init_end
    while i < n:
        ref_mean = np.mean(reference_buffer[-window_size:]) if len(reference_buffer) >= window_size else
np.mean(reference_buffer)
        delta = z_vals[i] - ref_mean

        # candidate jump?
        if abs(delta) >= thr_jump:
            direction = np.sign(delta) or 1.0
            jump_idx = i
            jump_val = z_vals[i]

            # search for a return within return_window_sec
            found_return = False
            j_limit = min(jump_idx + return_window_samples, n - 1)
            for j in range(jump_idx + 1, j_limit + 1):
                back_delta = z_vals[j] - jump_val
                if np.sign(back_delta) == -direction and abs(back_delta) >= thr_return:
                    # extend artifact block by ±1 s
                    s_ext = max(0, jump_idx - pad_samples)
                    e_ext = min(n - 1, j + pad_samples)

                    artifact_mask[s_ext:e_ext + 1] = True
                    artifact_events.append((s_ext, e_ext))

                    # continue after the extended block
                    i = e_ext + 1
                    found_return = True
                    break

            if not found_return:
                reference_buffer.append(z_vals[i])
                i += 1
        else:
            reference_buffer.append(z_vals[i])
            i += 1

    return artifact_mask, artifact_events



def interpolate_artifacts(raw_vals, artifact_mask=None, artifact_events=None):
    """
    Linearly interpolate artifact regions in the RAW EDA array (µS).

    - Left anchor: last non-artifact sample before the block
    - Right anchor: first non-artifact sample after the block (raw value)
    - Signal boundaries: if only one anchor is available, fill the entire
      block with that anchor value

    Returns
    -------
    corrected_raw_vals : np.ndarray
        Copy of raw_vals with linear interpolation across artifact samples.
    """
    x = np.asarray(raw_vals, dtype=float)
    n = len(x)
    if n == 0:
```

```
    return x.copy()

# --- construct boolean mask ---
if artifact_mask is None:
    mask = np.zeros(n, dtype=bool)
    if artifact_events:
        for s, e in artifact_events:
            s = max(0, int(s))
            e = min(n - 1, int(e))   # e is inclusive
            if s <= e:
                mask[s:e + 1] = True
else:
    mask = np.asarray(artifact_mask, dtype=bool)
    if mask.size != n:
        raise ValueError("artifact_mask length does not match raw_vals.")

if not mask.any():
    return x.copy()

corrected = x.copy()
keep = ~mask
if keep.sum() == 0:
    return corrected  # safety: all samples are artifacts, so leave unchanged

# --- identify contiguous blocks ---
# starts: transition F->T; ends: transition T->F
starts = np.where(~mask[:-1] & mask[1:])[0] + 1
ends   = np.where(mask[:-1] & ~mask[1:])[0]

# also include the beginning and end of the signal
if mask[0]:
    starts = np.r_[0, starts]
if mask[-1]:
    ends = np.r_[ends, n - 1]


for s, e in zip(starts, ends):
    # indices of clean samples before and after the block
    left_clean_idx  = np.where(keep[:s])[0]
    right_clean_idx = np.where(keep[e + 1:])[0] + (e + 1)

    # left/right anchors as actual clean points (index + value)
    left_anchor = right_anchor = None
    left_pos = right_pos = None

    if left_clean_idx.size > 0:
        left_pos = int(left_clean_idx[-1])  # last clean index before s
        left_anchor = float(x[left_pos])  # value at that index

    if right_clean_idx.size > 0:
        right_pos = int(right_clean_idx[0])  # first clean index after e
        right_anchor = float(x[right_pos])  # value at that index

    if left_anchor is None and right_anchor is None:
        continue
    elif left_anchor is None:
        corrected[s:e + 1] = right_anchor
        continue
    elif right_anchor is None:
        corrected[s:e + 1] = left_anchor
        continue

    # straight line between the clean anchors (left_pos → right_pos),
    # while overwriting only the artifact samples (s..e)
    xi = np.arange(s, e + 1)
    corrected[xi] = np.interp(
        xi,
        np.array([left_pos, right_pos], dtype=float),
        np.array([left_anchor, right_anchor], dtype=float),
    )

return corrected
```
