# Supplementary material for: Validity Assessment of Wrist-Worn Electrodermal Activity Measurement During Relaxation and Acute Stressor Tasks: Comparing the EmbracePlus and Shimmer3 GSR+
Source: Sensors (Basel). 2026 Oct 27;26(21):6795. doi: 10.3390/s26216795 (PMC13765827; doi:10.3390/s26216795)

Supplementary File S2: Distributions of inter-device differences at the parameter level

Histograms and boxplots show the distributions of inter-device differences (Shimmer3 GSR+ minus EmbracePlus) for (A, E) mean phasic activity, (B, F) mean SCL, (C, G) summed SCR amplitude/min, and (D, H) SCR count/min across 41 participants. Figures A−D show the differences on the original measurement scale, whereas Figures E−H show the corresponding differences after LN(x+1) transformation of the device-specific values. Each histogram illustrates the frequency distribution of inter-device differences, while the corresponding boxplot shows the median, interquartile range, and potential outliers.

(A) Mean phasic (µS):

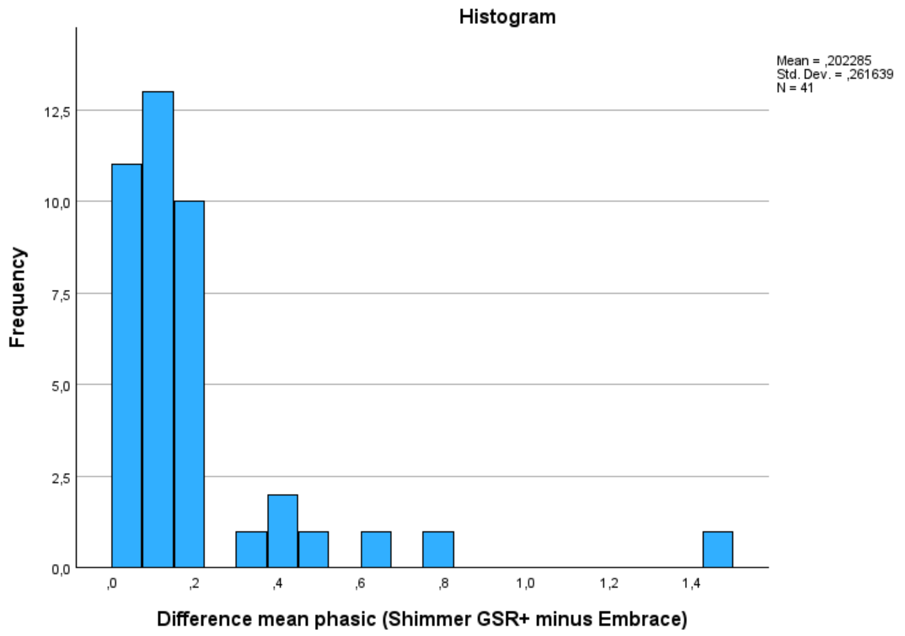

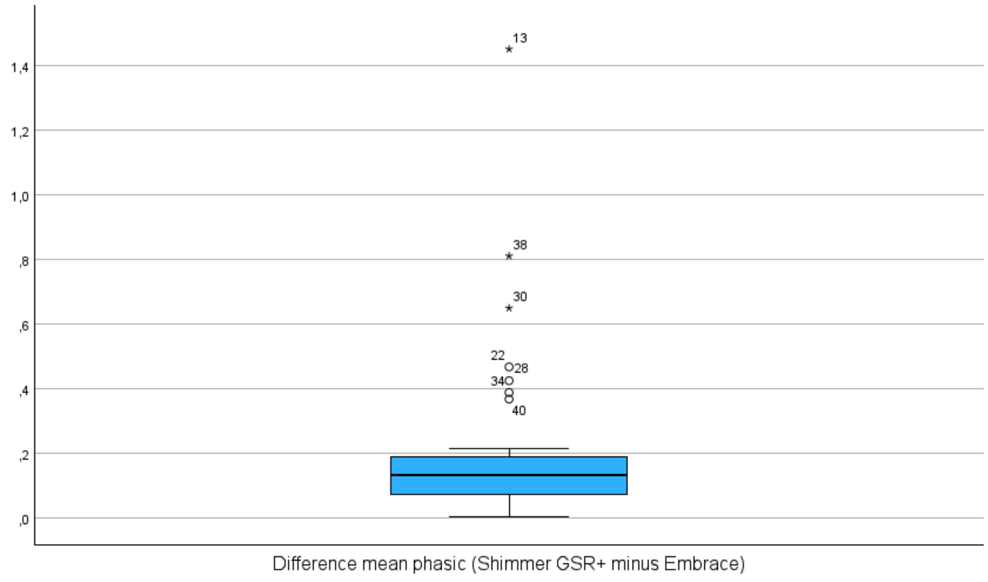

(B) Mean SCL (µS) :

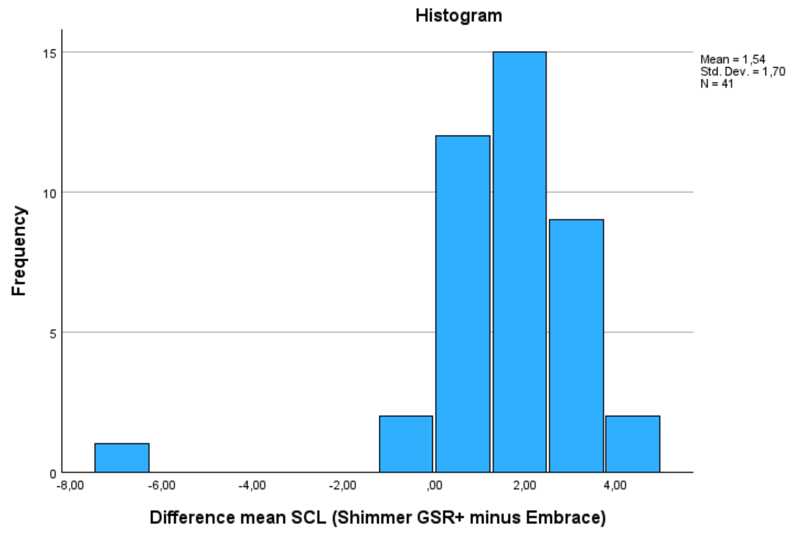
Histogram
Mean = 1,54
Std. Dev. = 1,70
N = 41
Frequency
15
10
5
0
-8,00
-6,00
-4,00
-2,00
,00
2,00
4,00
Difference mean SCL (Shimmer GSR+ minus Embrace)

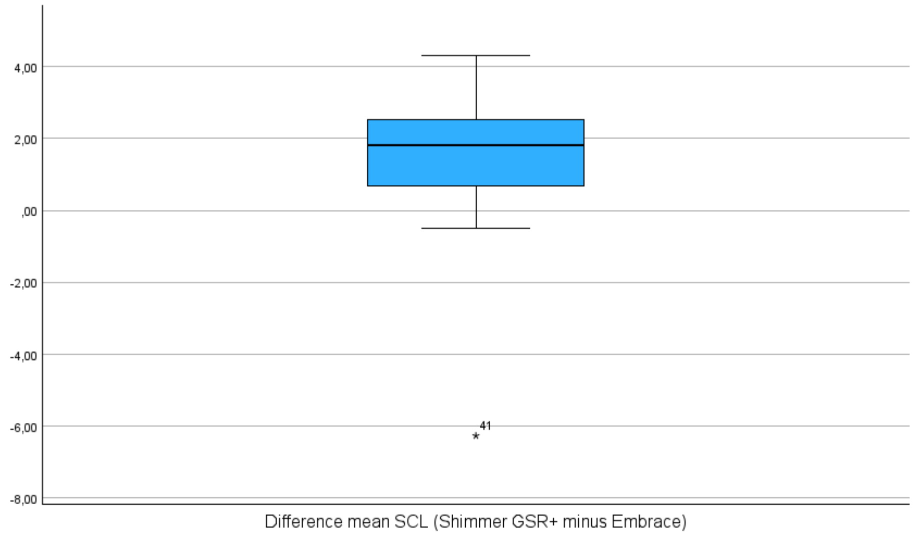
4,00
2,00
,00
-2,00
-4,00
-6,00
-8,00
41
Difference mean SCL (Shimmer GSR+ minus Embrace)

(C) Summed SCR amplitude/min (µS/min)

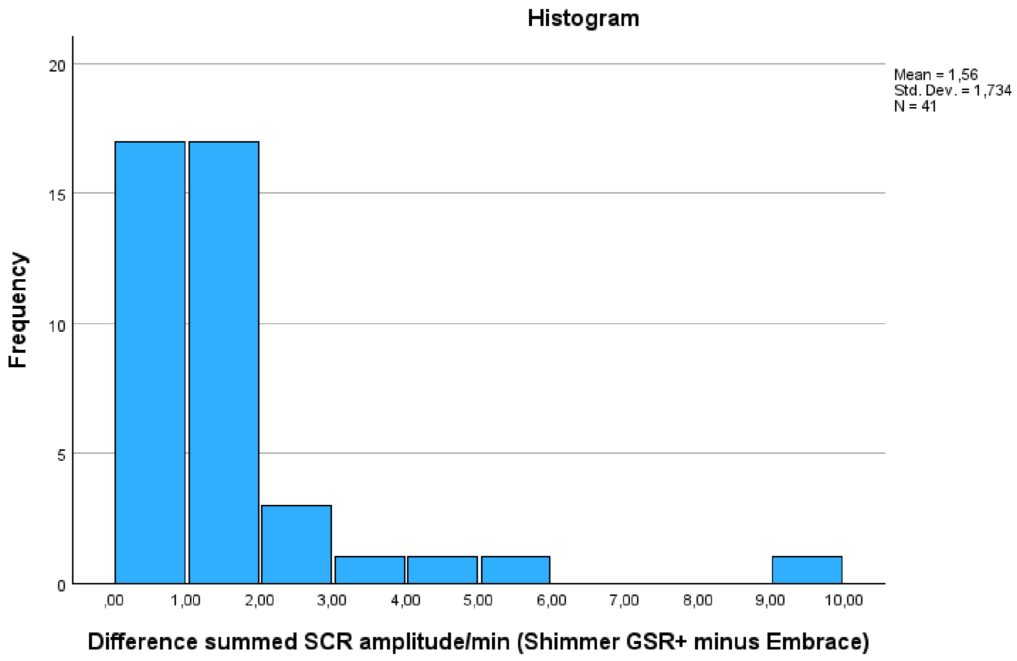

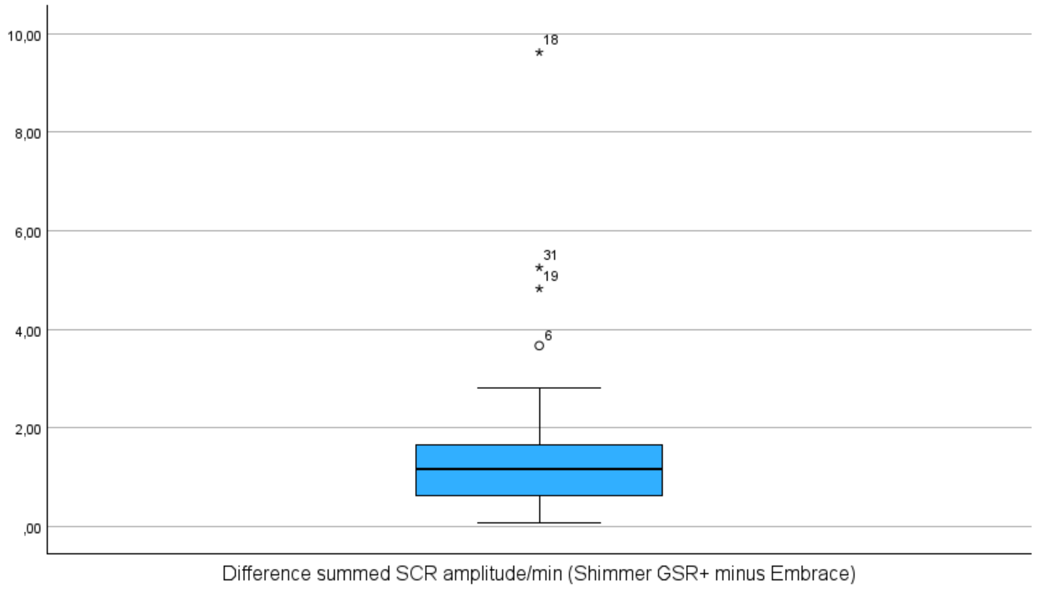

(D) SCR Count/min

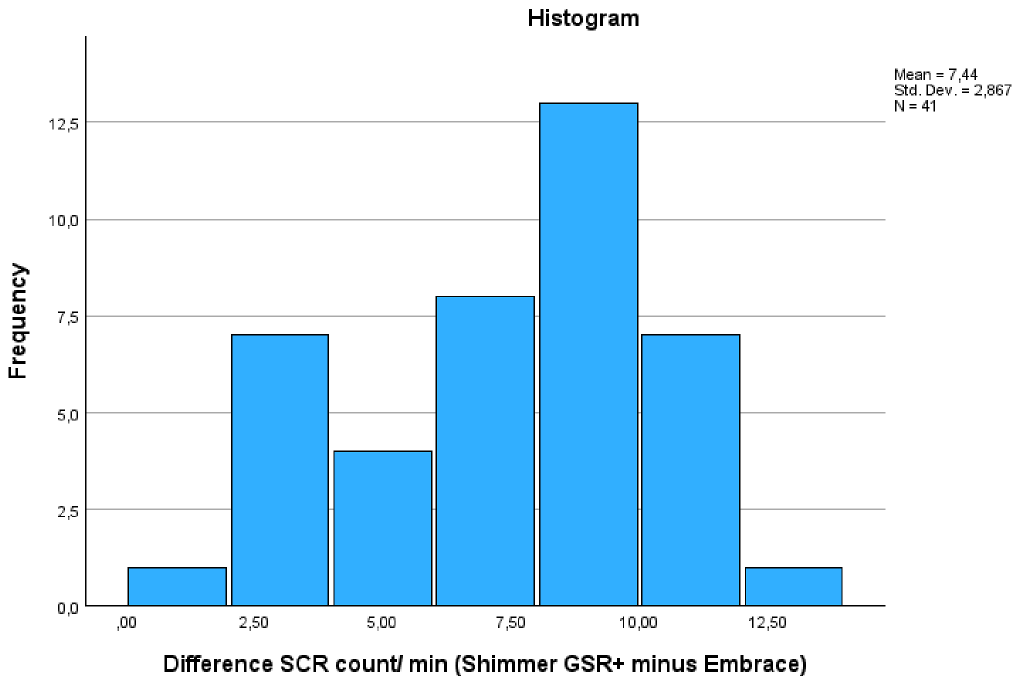

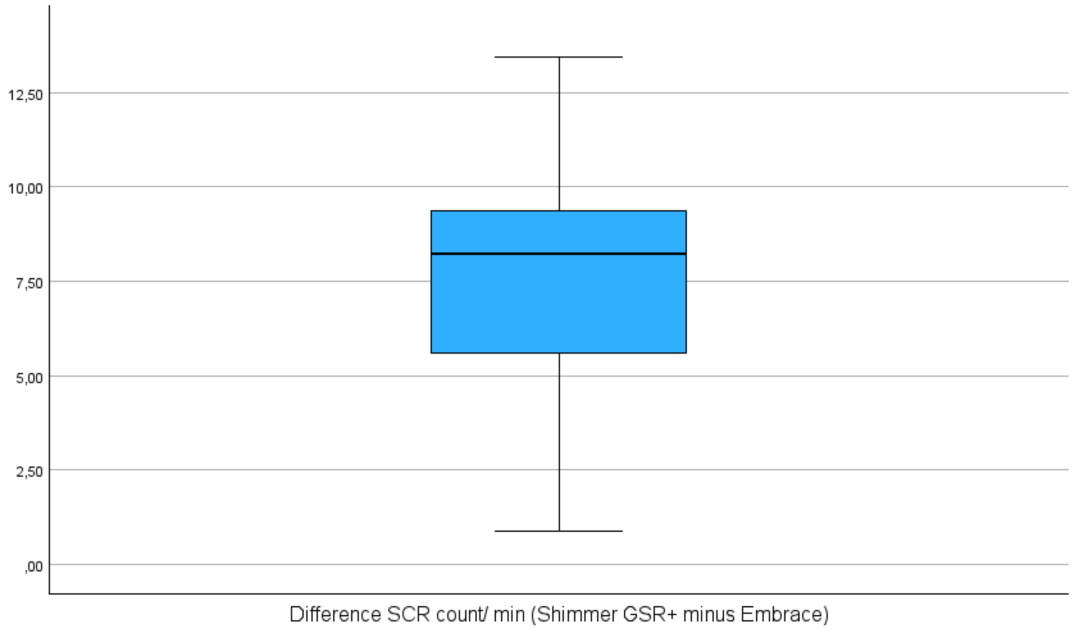

(E) Mean phasic [LN(X+1)]

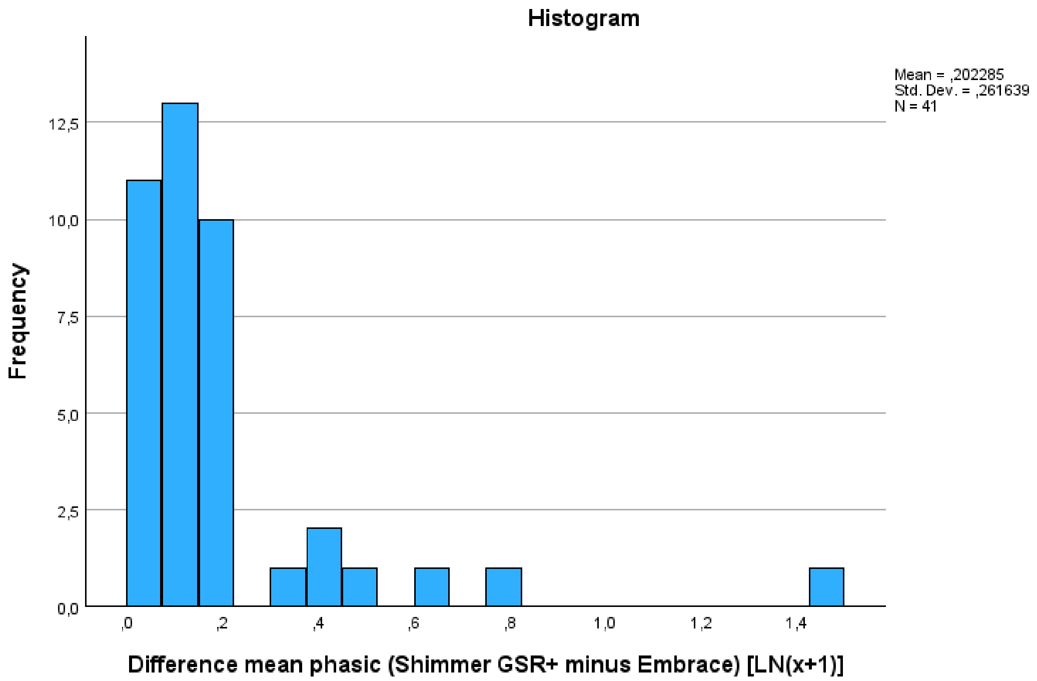

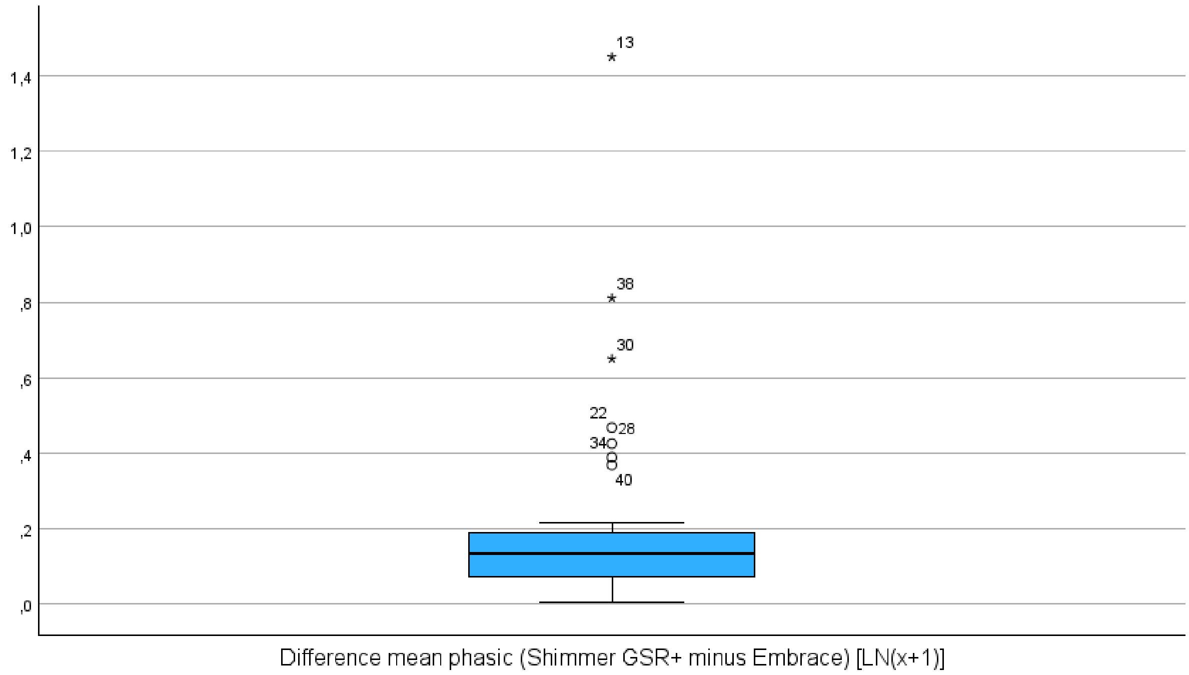

(F) Mean SCL [LN(X+1)]

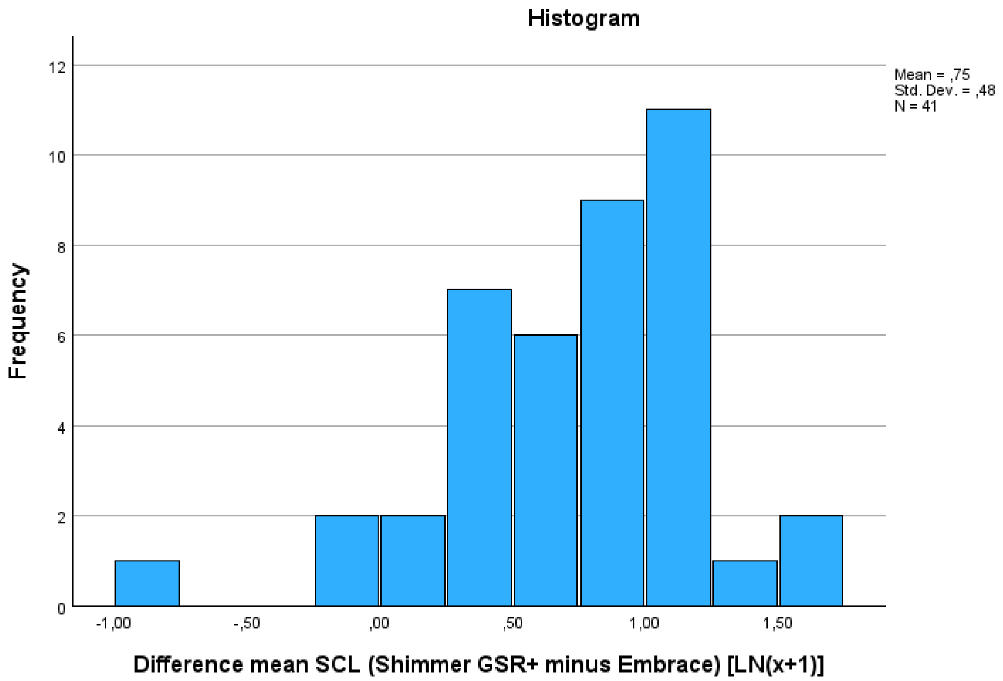

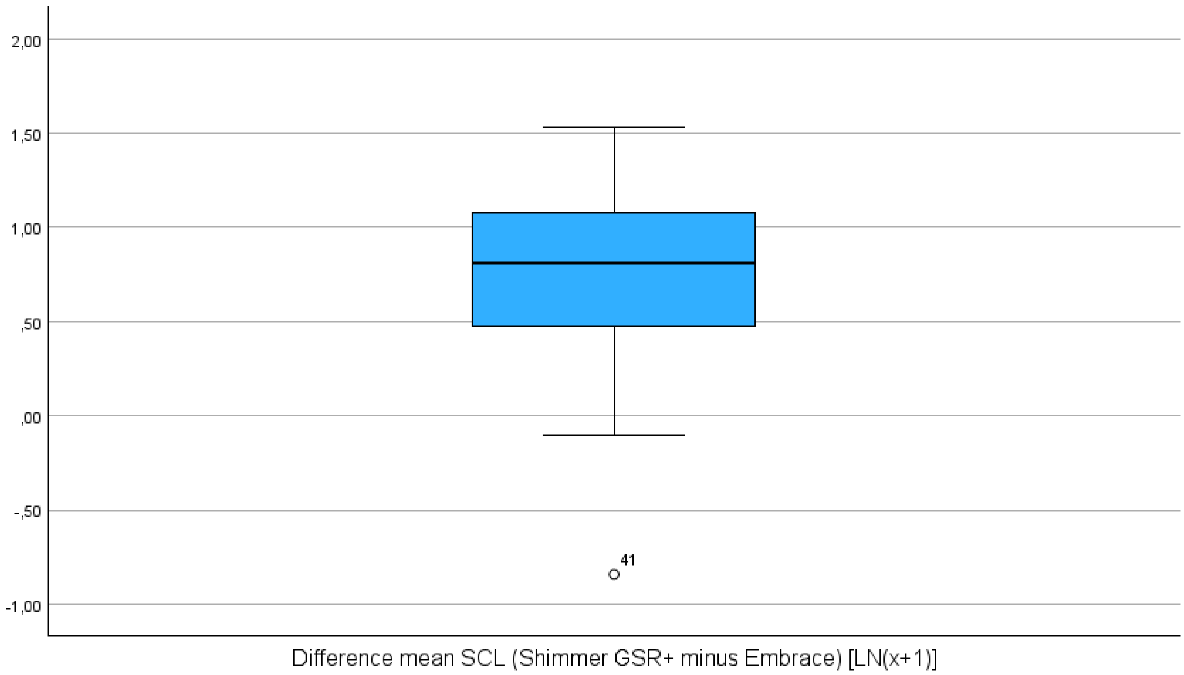

(G) Summed SCR Amplitude/min [LN(X+1)]

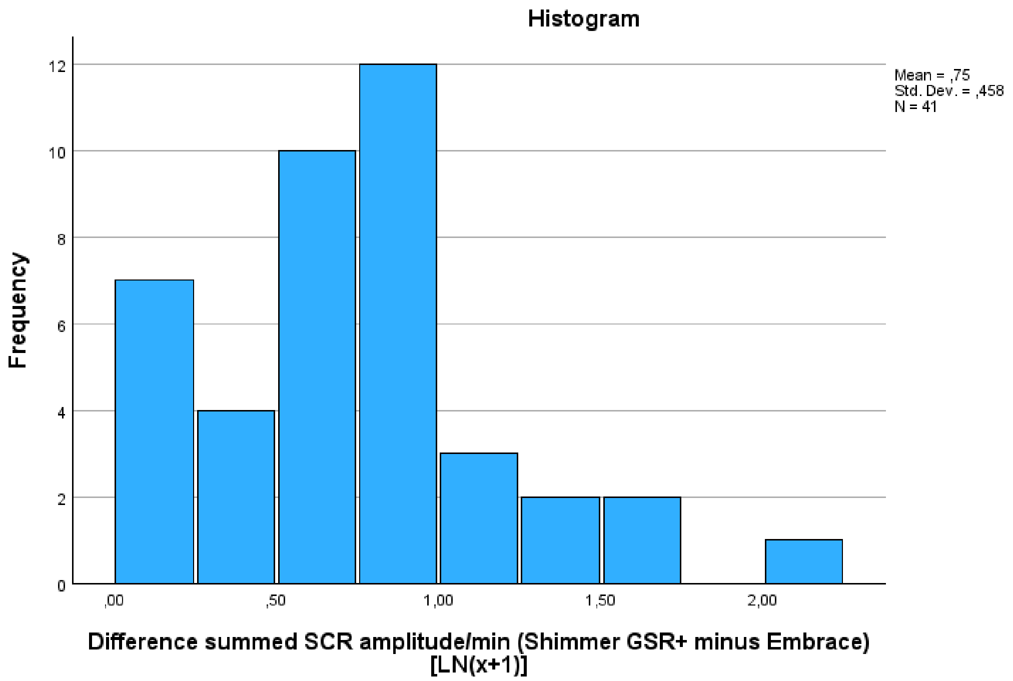

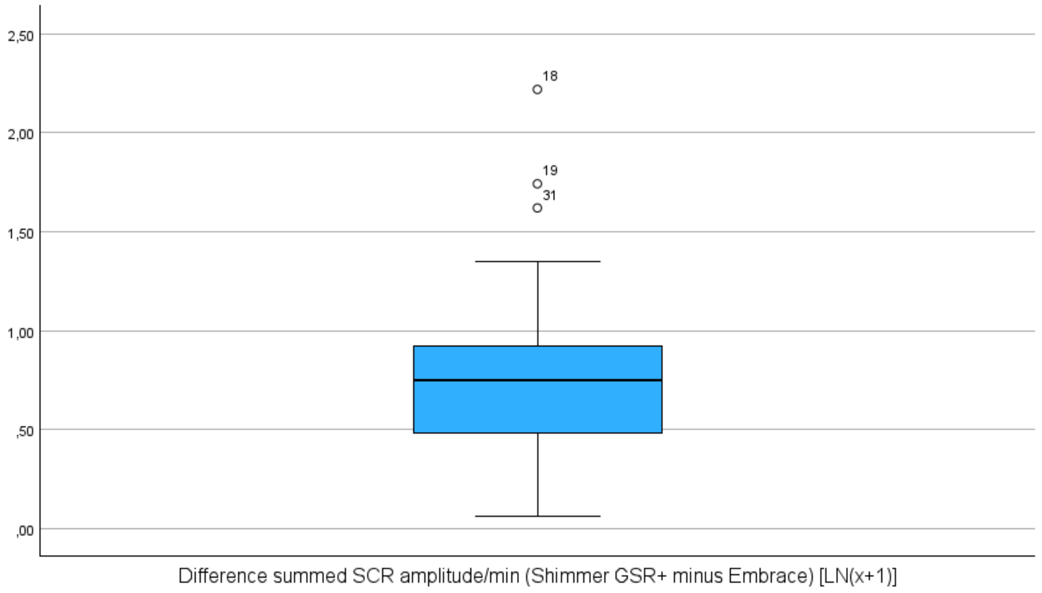

(H) SCR Count/min [LN(X+1)]

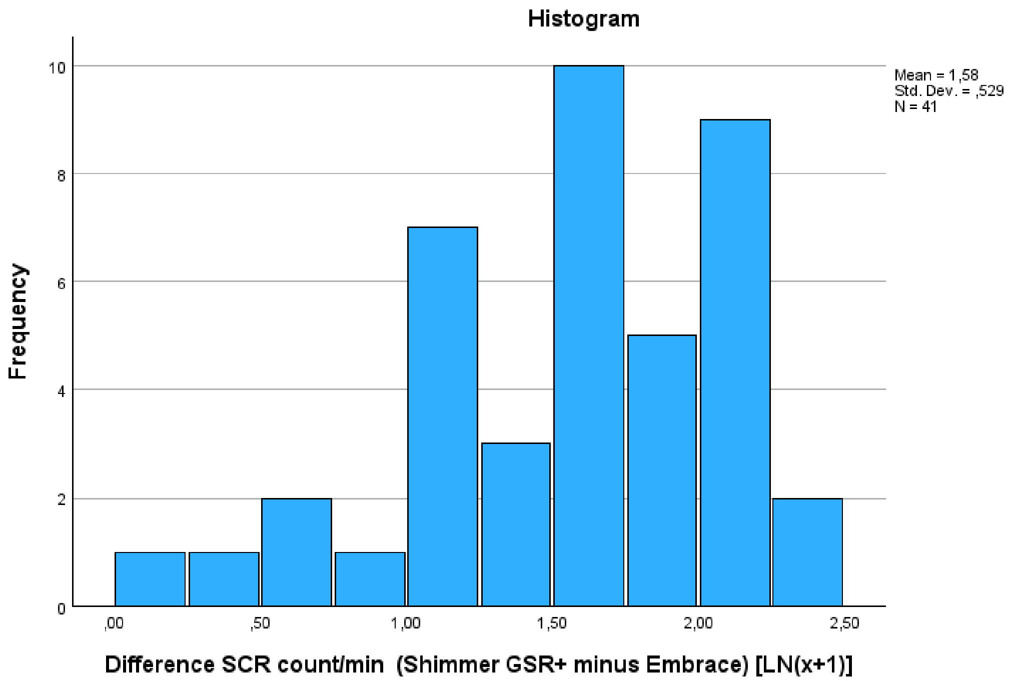

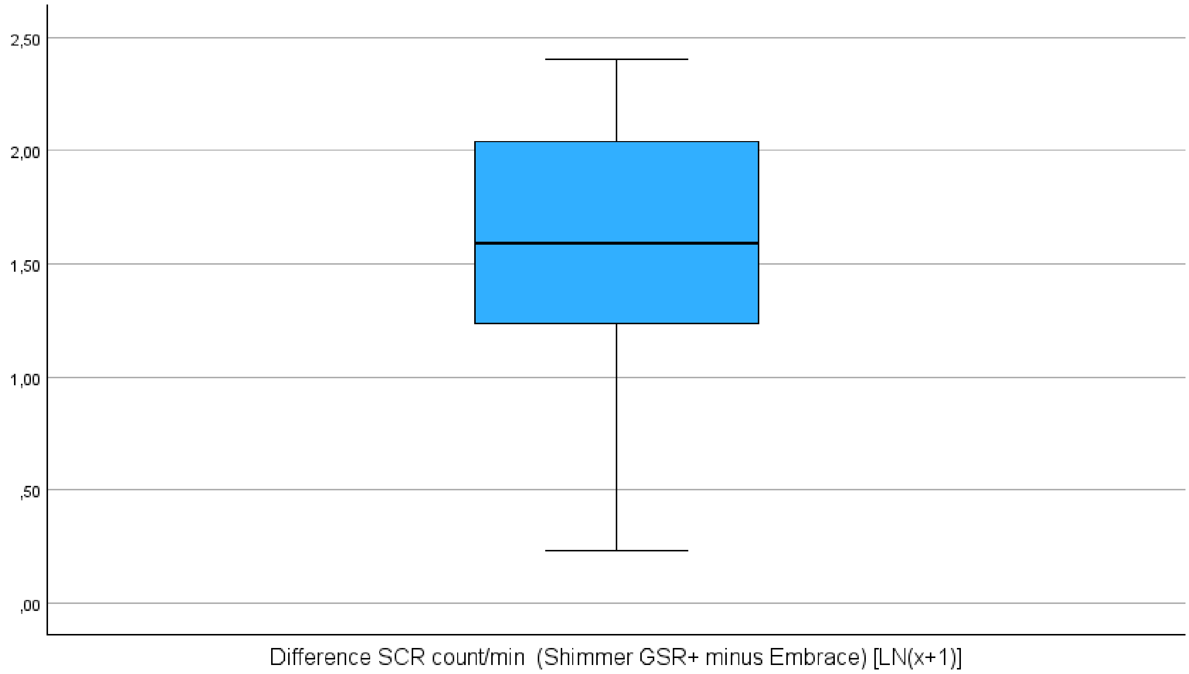

Supplement: Supplementary file 1 [file sensors-26-06795-s001.zip › Supplementary File S2 Distribution inter-device parameter.pdf]
