# Supplementary material for: Validity Assessment of Wrist-Worn Electrodermal Activity Measurement During Relaxation and Acute Stressor Tasks: Comparing the EmbracePlus and Shimmer3 GSR+
Source: Sensors (Basel). 2026 Oct 27;26(21):6795. doi: 10.3390/s26216795 (PMC13765827; doi:10.3390/s26216795)

Supplementary File S3: Boxplots of EDA metrics by event and device

The boxplots of the EDA metrics are shown on the original scale (A-D) and the LN(x+1)-scaled -transformed scale (E-H):

A) Mean phasic (original scale)

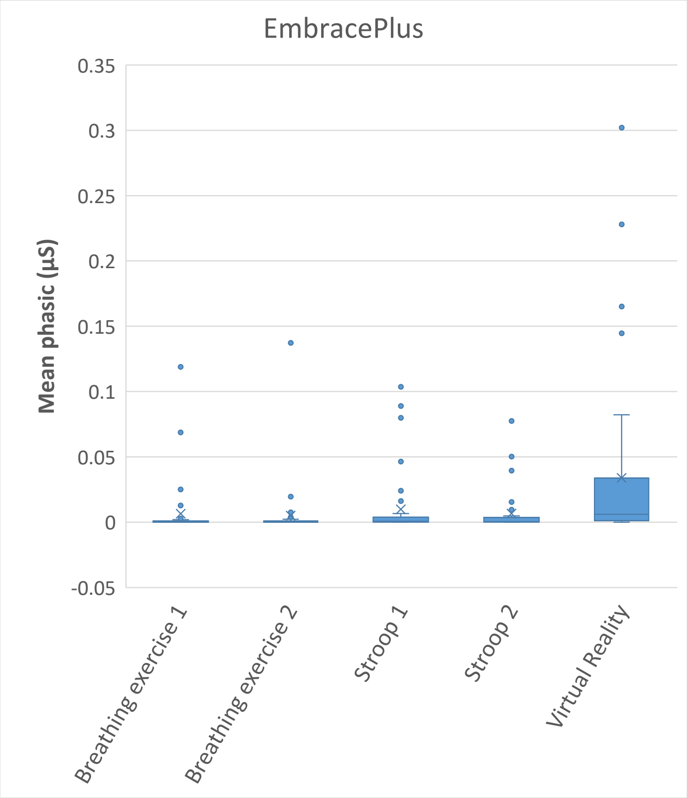

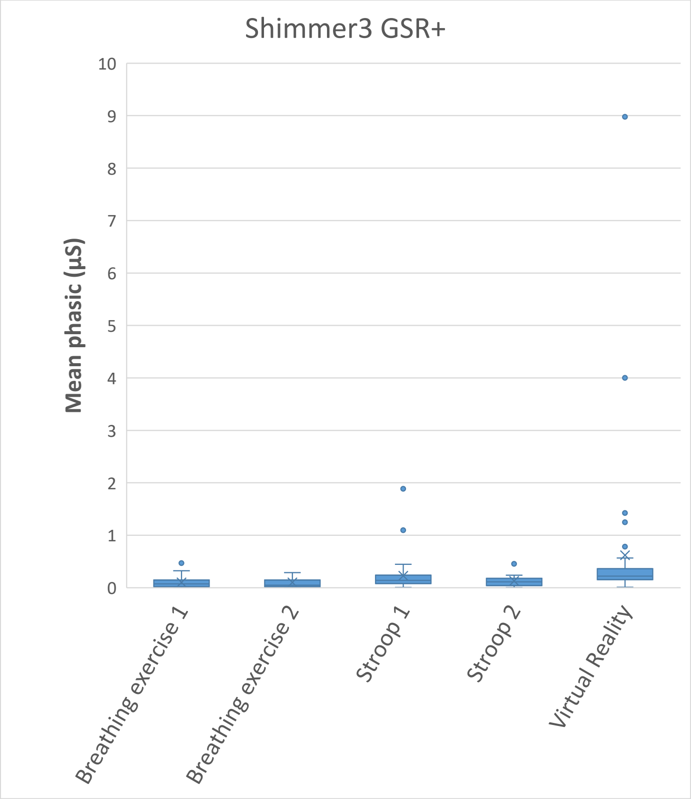

B) Mean SCL (original scale)

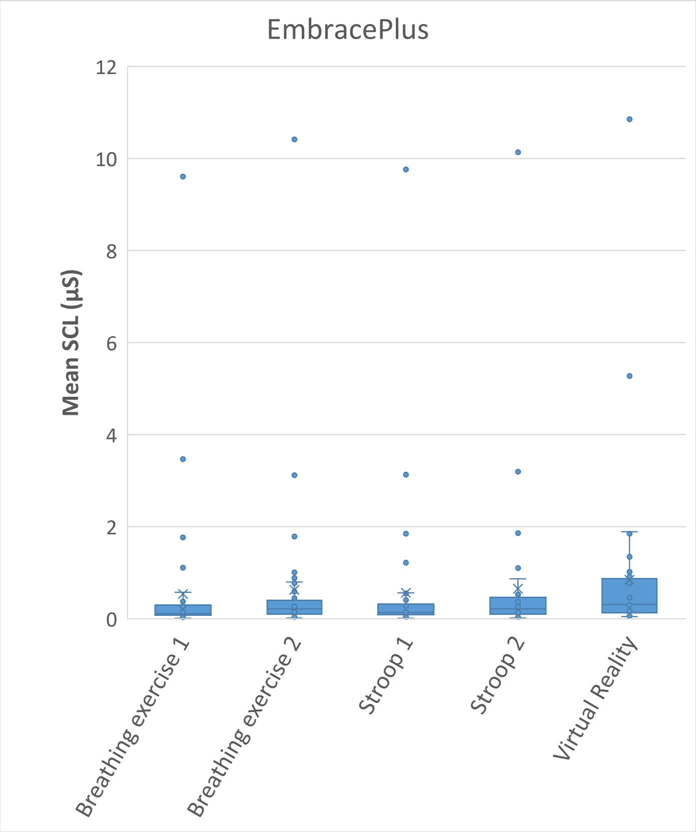

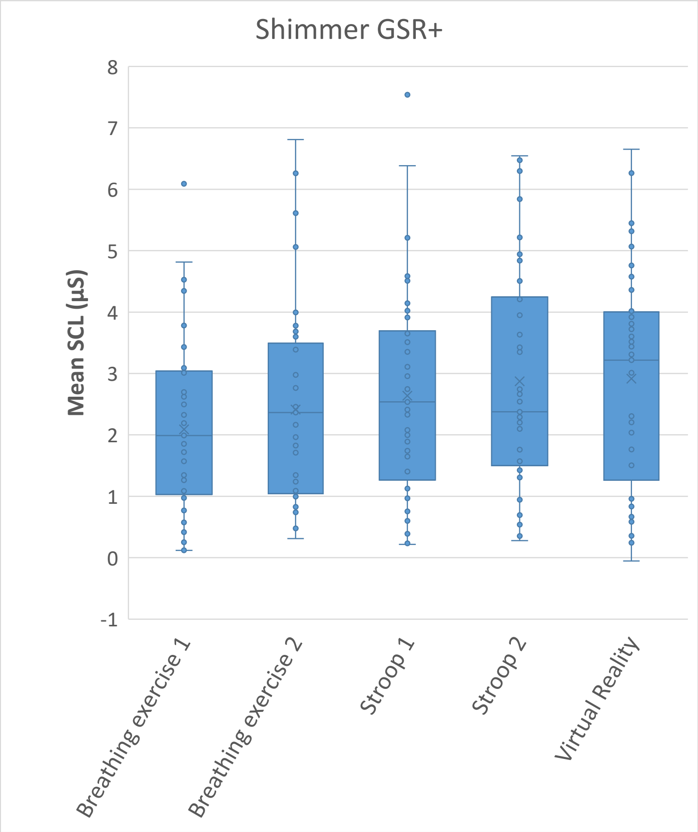

C) Summed SCR amplitude/min (original scale)

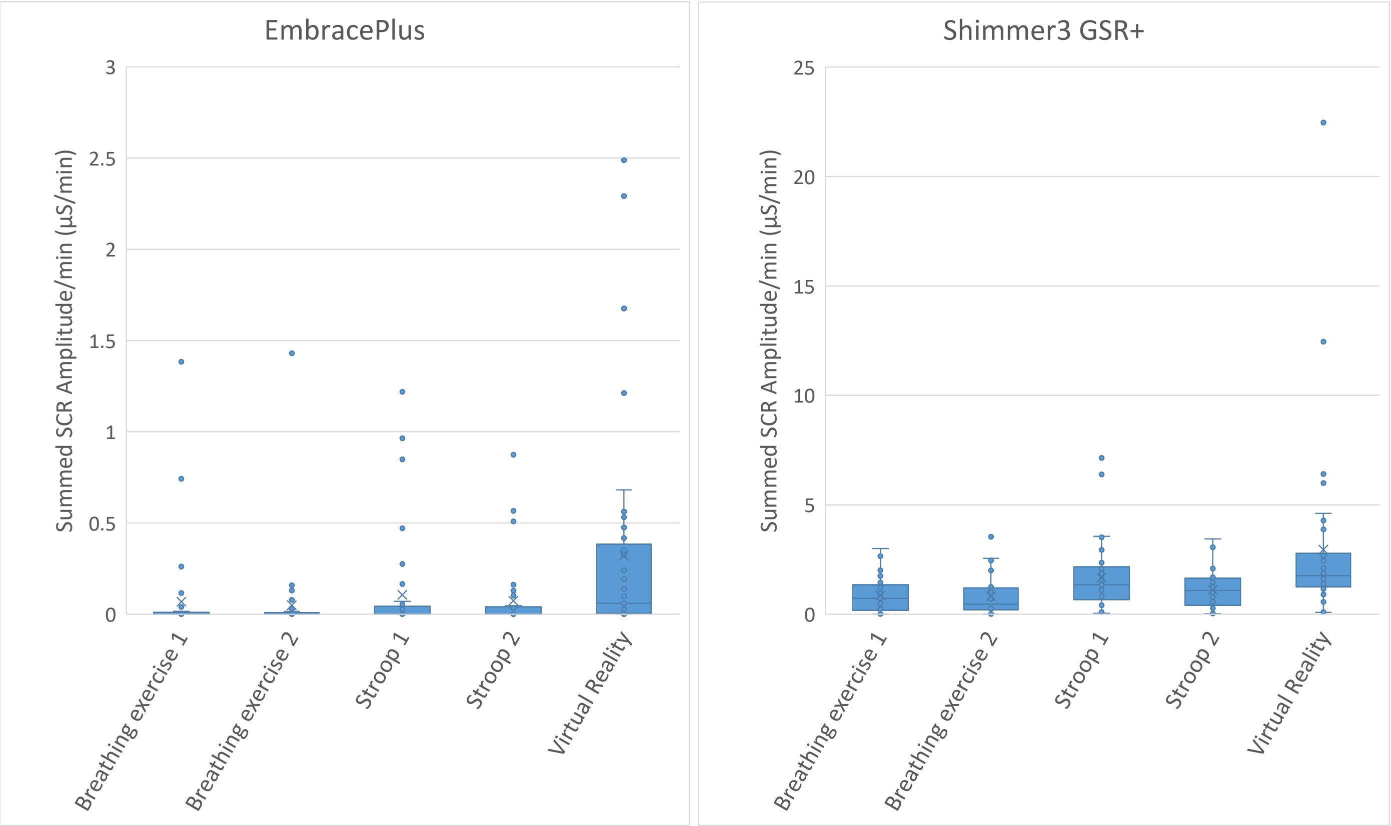

D) SCR count/min (original scale)

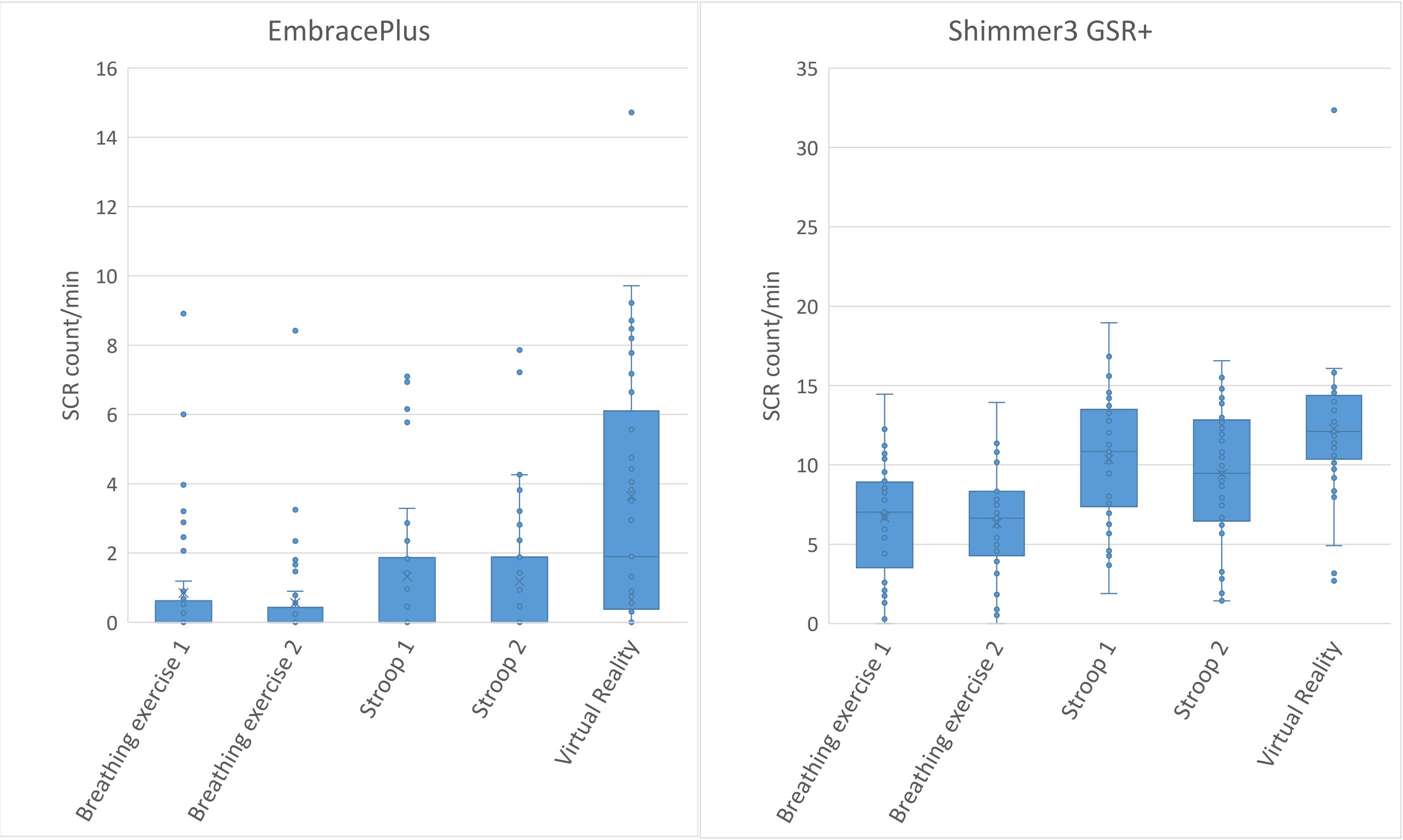

E) Mean phasic (LN(X+1))

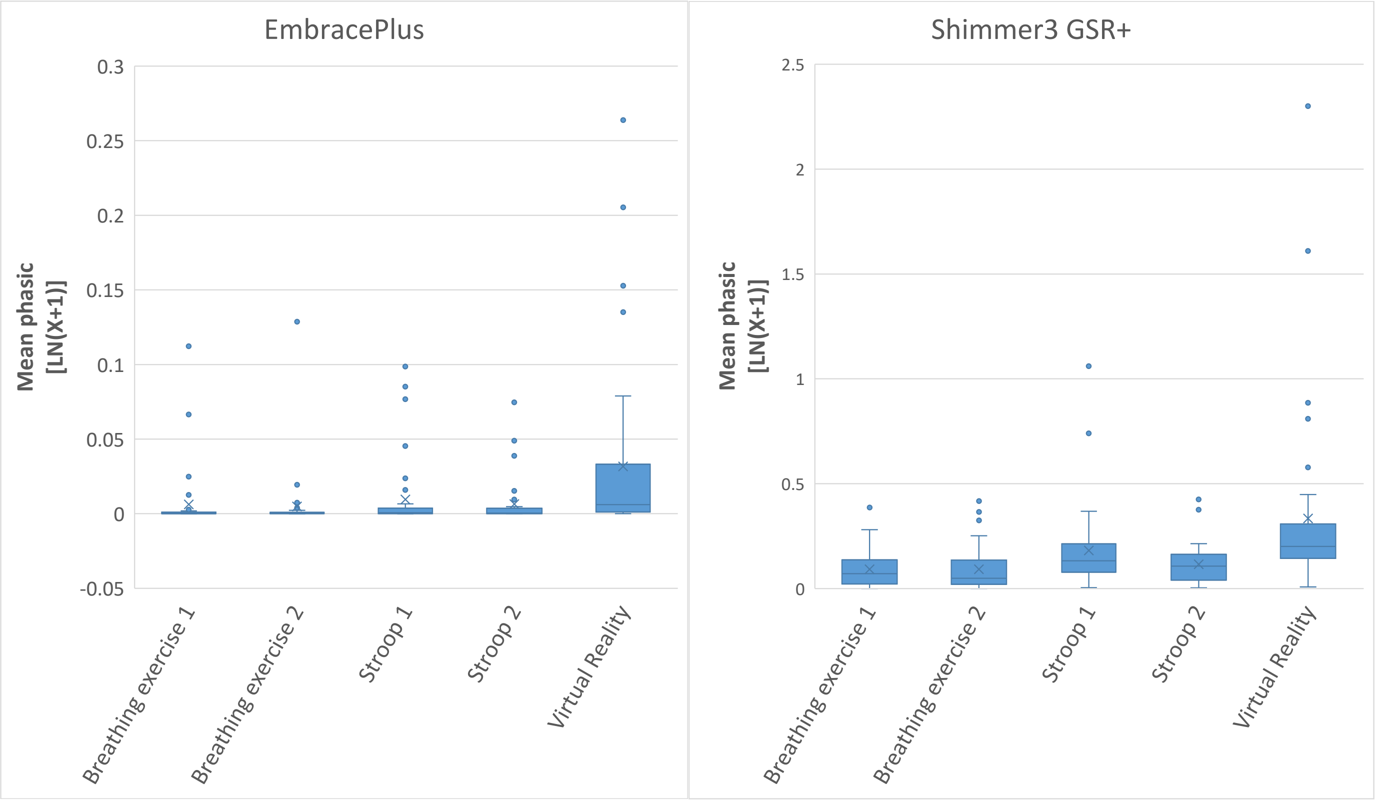

F) Mean SCL (LN(X+1))

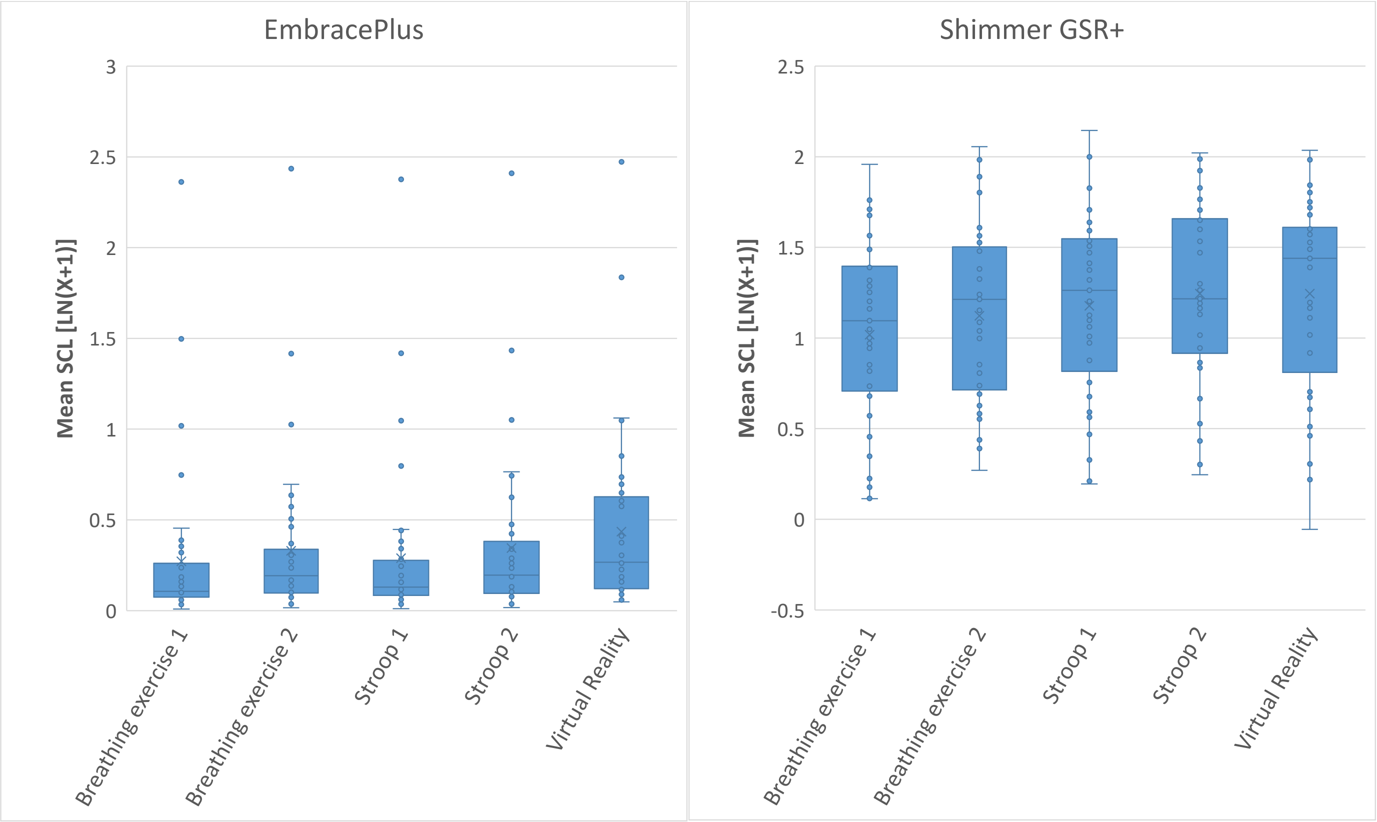

G) Summed SCR amplitude/min (LN(X+1))

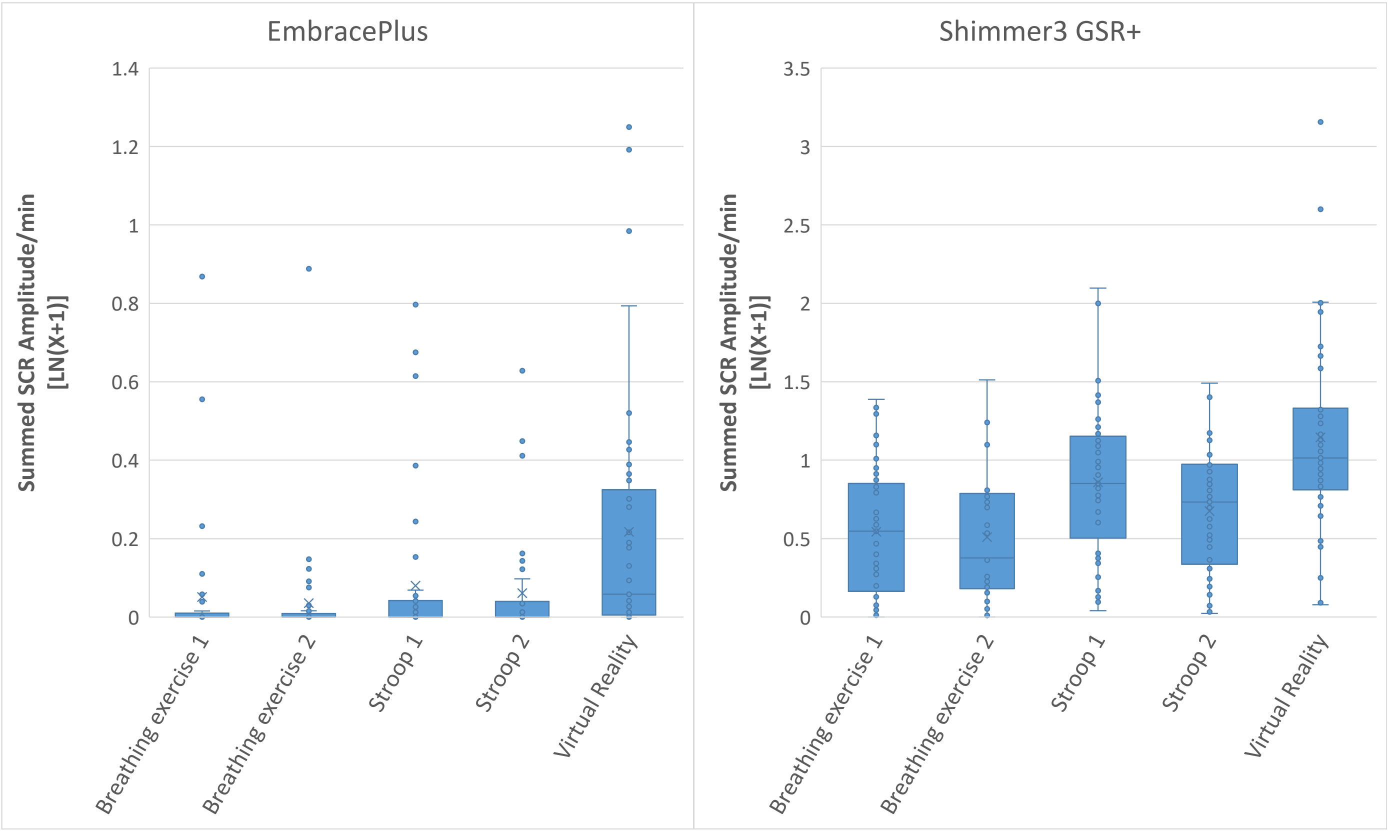

H) SCR Count/min (LN(X+1))

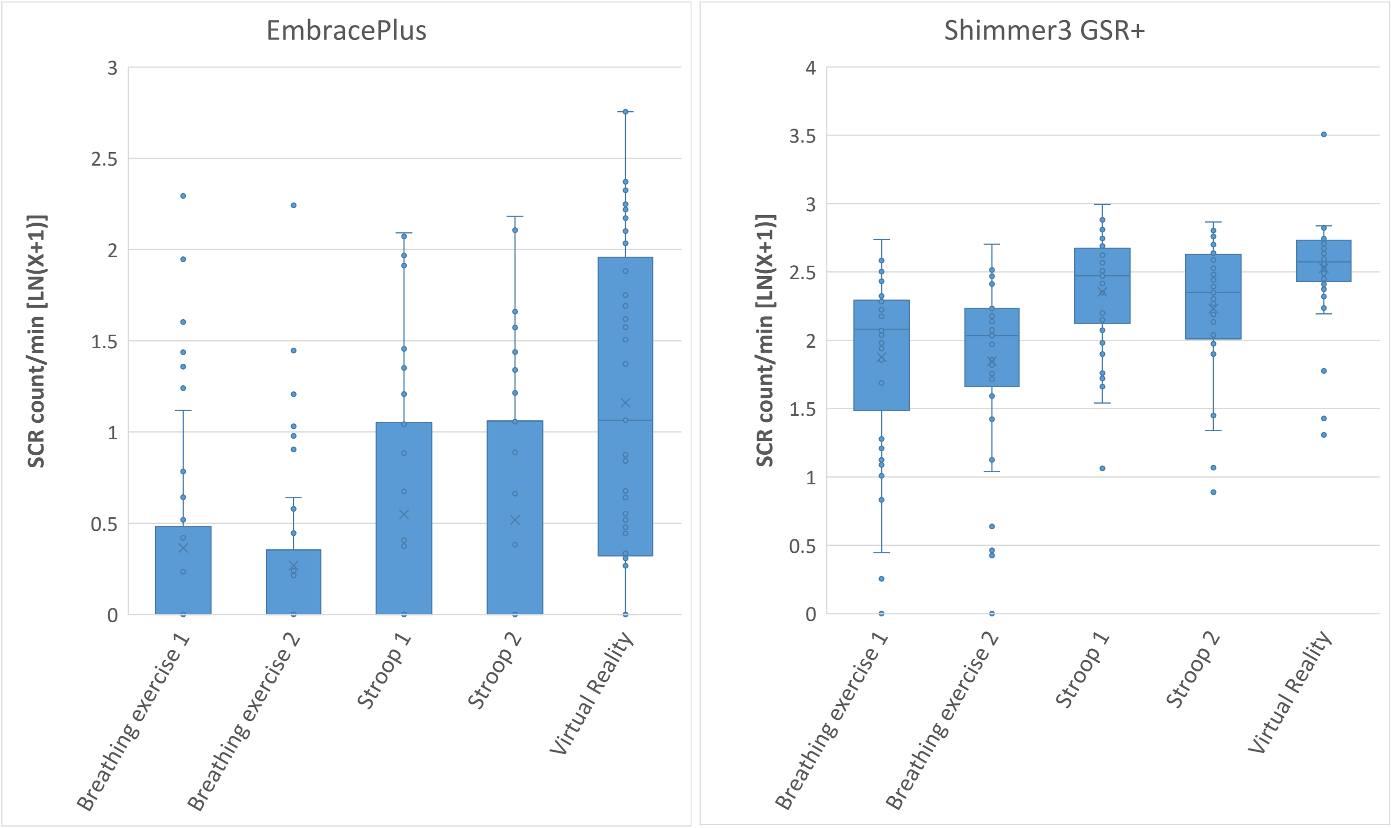

Supplement: Supplementary file 1 [file sensors-26-06795-s001.zip › Supplementary File S3 Event-device Boxplots.pdf]
