# Supplementary material for: Validity Assessment of Wrist-Worn Electrodermal Activity Measurement During Relaxation and Acute Stressor Tasks: Comparing the EmbracePlus and Shimmer3 GSR+
Source: Sensors (Basel). 2026 Oct 27;26(21):6795. doi: 10.3390/s26216795 (PMC13765827; doi:10.3390/s26216795)
Supplement: Supplementary file 1 [file sensors-26-06795-s001.zip › Supplementary File S4 LN(x+1)-scaled bland altman plots.pdf]

Supplementary File S4: LN(x + 1)-scaled Bland–Altman plots

Bland–Altman plots of parameter-level agreement between devices on the LN(x+1)-transformed scale. Bland–Altman plots are shown for A) mean phasic, B) mean SCL, C) summed SCR amplitude/minute, and D) SCR count/min. Device-specific values were transformed using LN(x+1) prior to calculating the between-device mean and difference. Differences were calculated as (LN[Shimmer3 GSR+ + 1] − LN[EmbracePlus + 1]). The black solid line indicates the mean bias, the red dashed lines indicate the 95% limits of agreement (bias ± 1.96 SD), and the green lines indicate the transformed *a priori* acceptable boundaries. No *a priori* acceptable boundary was available for total mean phasic.

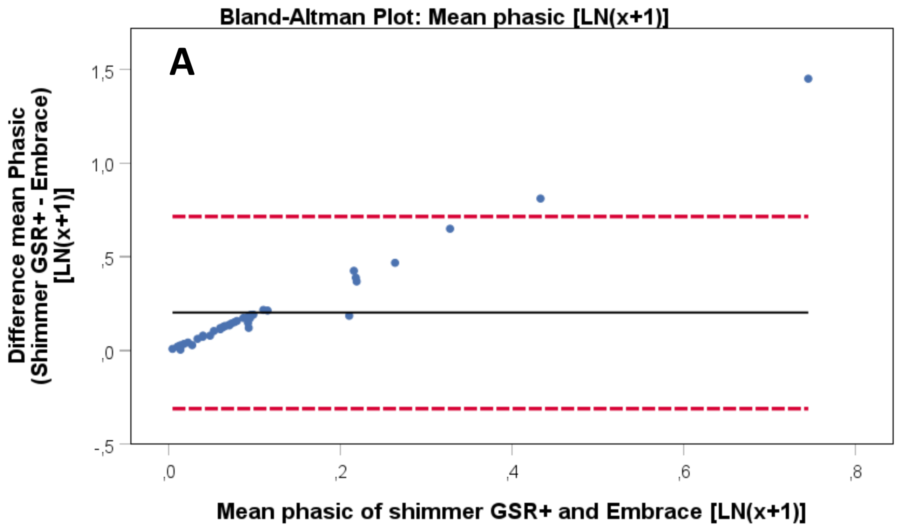

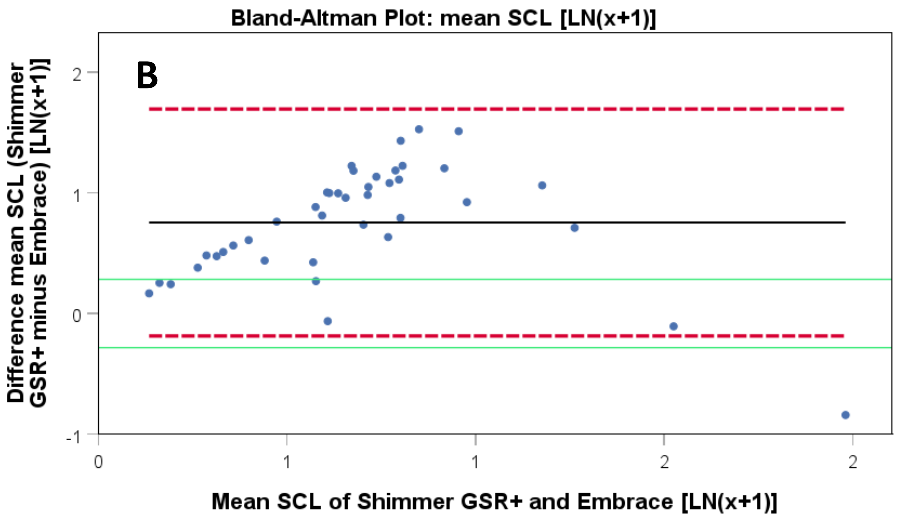

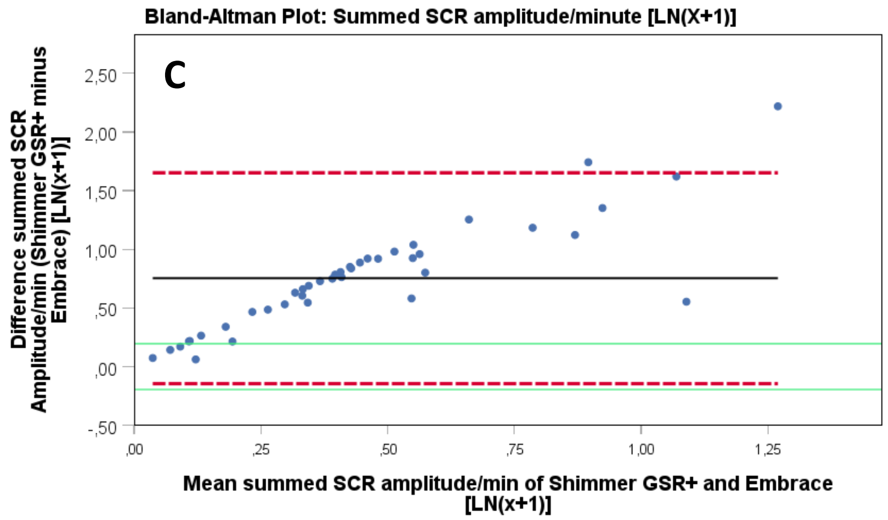

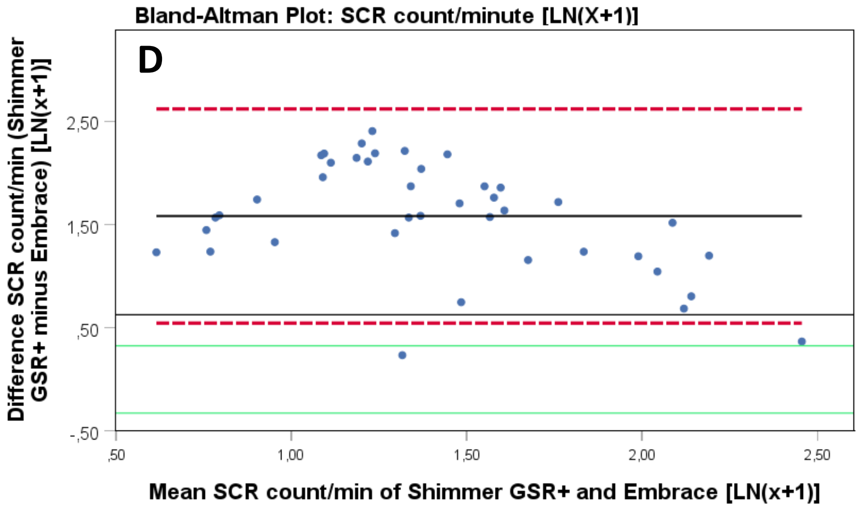

Table S1. Proportional bias was assessed using linear regression on LN(x + 1)-transformed device-specific values, with the inter-device difference (LN[Shimmer3 GSR+ + 1] − LN[EmbracePlus + 1]) as the dependent variable and the mean of the two transformed device values as the independent variable. Primary analyses included all valid participants (n = 41). Where the maximum Cook's distance exceeded 1, a sensitivity analysis was performed after exclusion of the single most influential observation. B represents the unstandardized regression coefficient estimated from the original sample; a positive B indicates that the inter-device difference increases with measurement magnitude, whereas a negative B indicates that the difference decreases with measurement magnitude. Bias-corrected and accelerated (BCa) 95% confidence intervals were based on 5000 bootstrap samples. BCa 95% confidence intervals excluding zero are shown in bold. N/A= Not Applicable.

LN(x + 1)-transformed EDA metric	Analysis	*n*	B	BCa 95% CI
Mean Phasic	Primary analysis	41	1.892	**[1.653; 1.944]**
	Sensitivity analysis	40	1.801	**[1.477; 1.933]**
Mean SCL	Primary analysis	41	−0.071	[−0.741; 1.396]
	Sensitivity analysis	40	0.524	[−0.165; 1.455]
SCR amplitude per minute	Primary analysis	41	1.359	**[0.864; 1.717]**
	Sensitivity analysis	40	1.584	**[1.390; 1.754]**
SCR count per minute	Primary analysis	41	−0.525	**[−0.892; −0.144]**
	Sensitivity analysis	N/A	N/A	N/A
