# Supplementary material for: Validity Assessment of Wrist-Worn Electrodermal Activity Measurement During Relaxation and Acute Stressor Tasks: Comparing the EmbracePlus and Shimmer3 GSR+
Source: Sensors (Basel). 2026 Oct 27;26(21):6795. doi: 10.3390/s26216795 (PMC13765827; doi:10.3390/s26216795)

Supplementary File S5: Event-level within-device plots for the EmbracePlus

Event-level within-device plots for the EmbracePlus. Plots showing (A, E) mean phasic activity, (B, F) mean SCL, (C, G) summed SCR amplitude/min, and (D, H) SCR count/min. Panels A–D show the original-scale data, whereas panels E–H show the corresponding LN(x+1)-transformed data. For each metric, the left panel shows the Stroop event set (Breathing Exercise 1 as baseline 1, Stroop I, Stroop II) and the right panel shows the VR event set (Breathing Exercise 2 as baseline 2, Virtual Reality). Grey lines represent individual participants, and the red line indicates the group mean ± SE. *statistically significant comparisons based on Bonferroni-corrected pairwise tests where applicable (p < .05).

A) Mean phasic

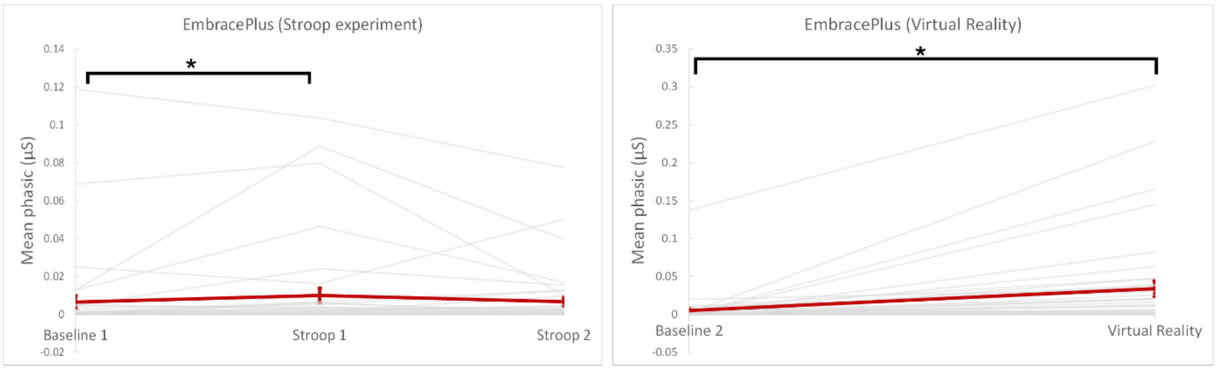

B) Mean SCL

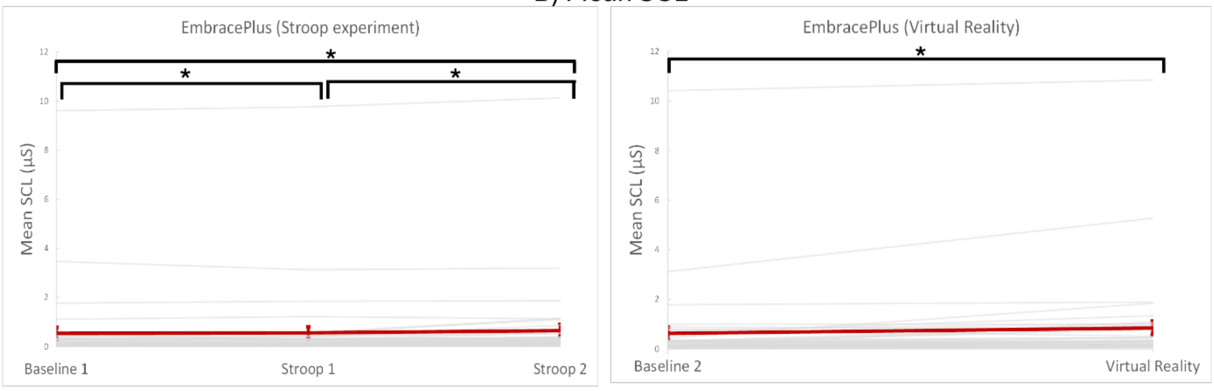

C) Summed SCR Amplitude/min

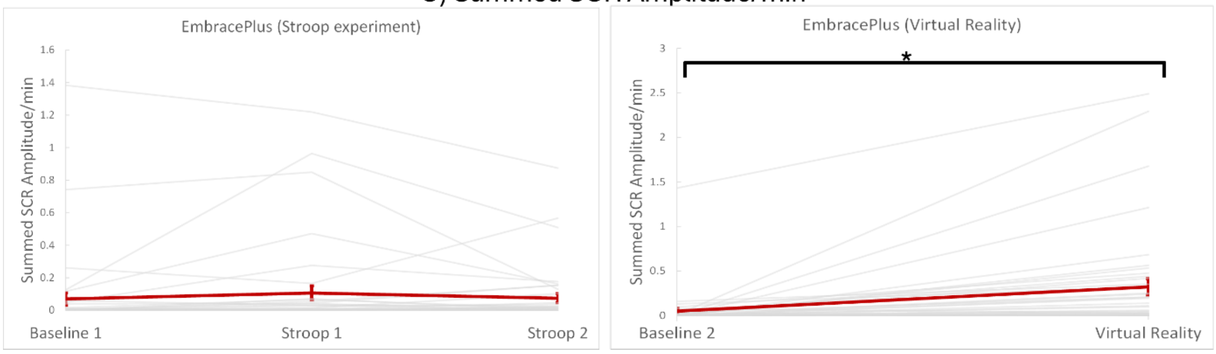

D) SCR Count/min

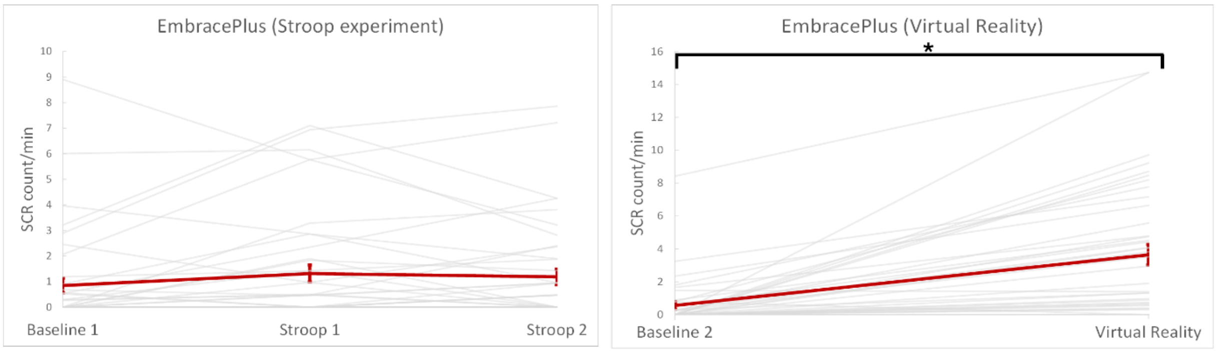

E) Mean phasic [LN(X+1)]

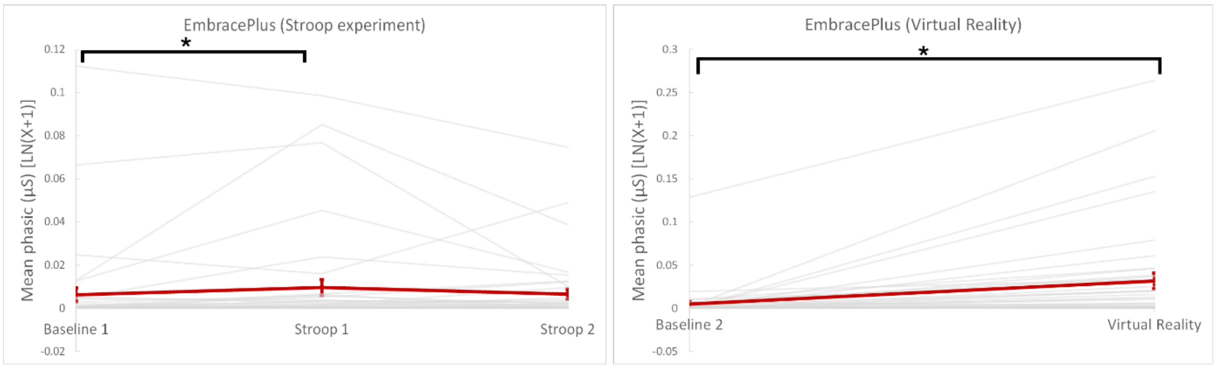

F) Mean SCL [LN(X+1)]

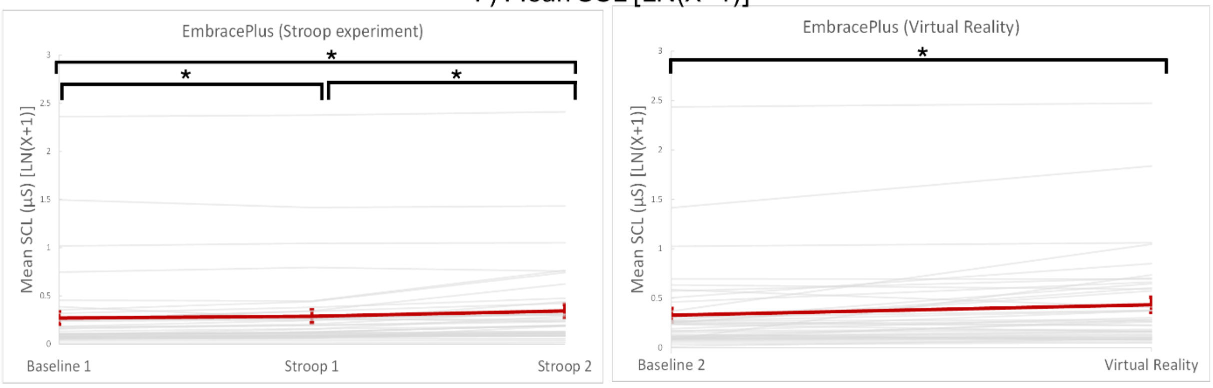

G) Summed SCR Amplitude/min [LN(x+1)]

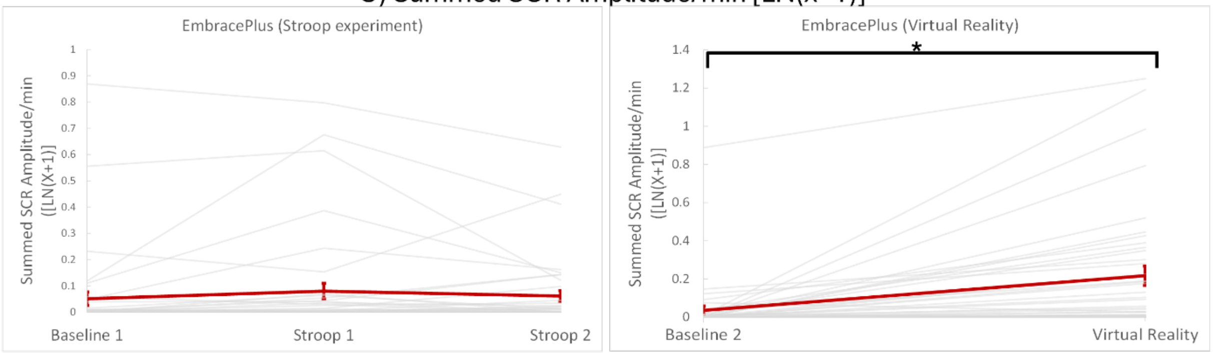

H) SCR Count/min [LN(x+1)]

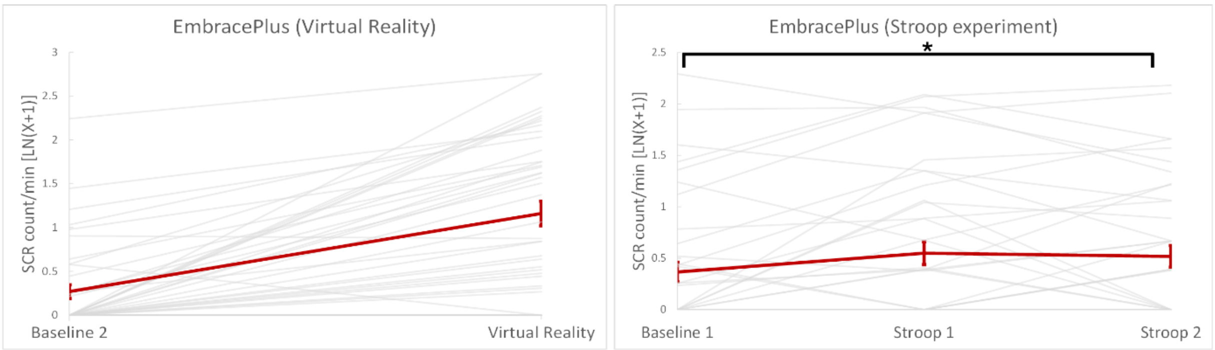

Supplement: Supplementary file 1 [file sensors-26-06795-s001.zip › Supplementary File S5 embrace events.pdf]
