# Supplementary material for: Validity Assessment of Wrist-Worn Electrodermal Activity Measurement During Relaxation and Acute Stressor Tasks: Comparing the EmbracePlus and Shimmer3 GSR+
Source: Sensors (Basel). 2026 Oct 27;26(21):6795. doi: 10.3390/s26216795 (PMC13765827; doi:10.3390/s26216795)

Supplementary File S7: LN(x + 1) scaled inter-device difference plots

LN(x+1) scaled inter-device difference plots. Plots show (A) mean phasic, (B) mean SCL, (C) summed SCR amplitude/min, and (D) SCR count/min, computed as Shimmer3 GSR+ minus EmbracePlus and displayed on the LN(x + 1) scale. For each metric, the left panel shows the Stroop event set (Breathing Exercise 1 as baseline 1, Stroop 1, Stroop 2) and the right panel shows the VR event set (Breathing Exercise 2 as baseline 2, Virtual Reality). Grey lines represent individual participants, and the red line indicates the group mean ± SE. Green horizontal lines indicate the *a priori* acceptability boundaries (±|reference effect|), shown on the same LN(x + 1) scale and derived from the corresponding Shimmer3 baseline-to-stressor change. For the Stroop event set, the reference effect was defined as the change from Breathing Exercise 1 to Stroop 1; for the VR event set, it was defined as the change from Breathing Exercise 2 to Virtual Reality.

A) Mean Phasic

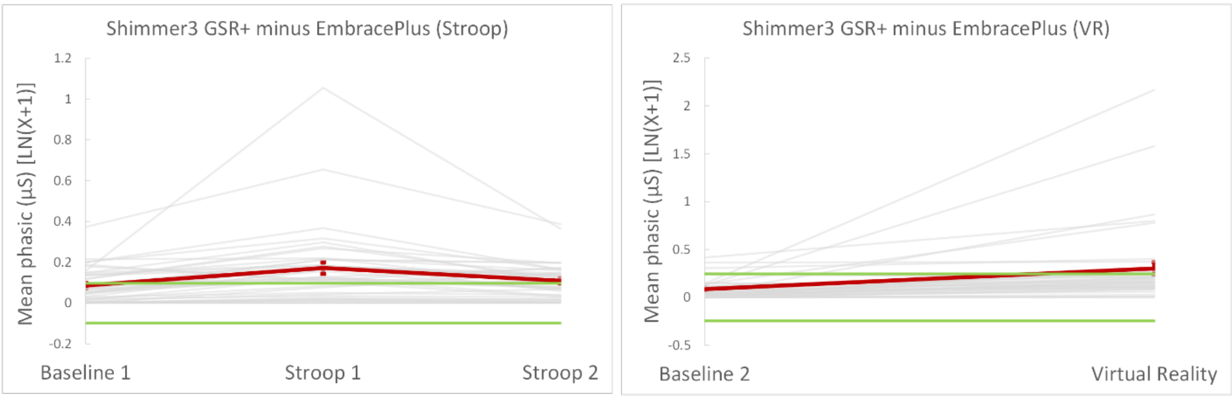

B) Mean SCL

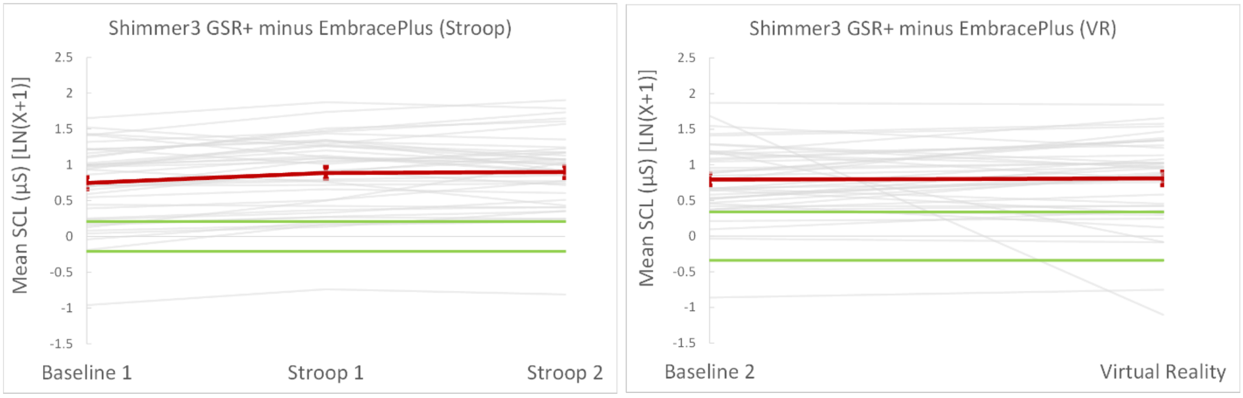

C) Summed SCR Amplitude/min

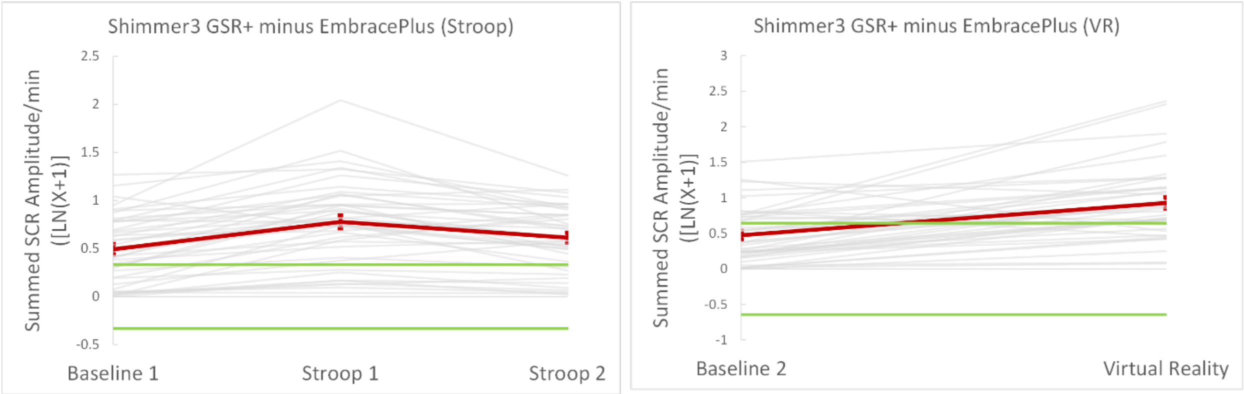

D) SCR Count/min

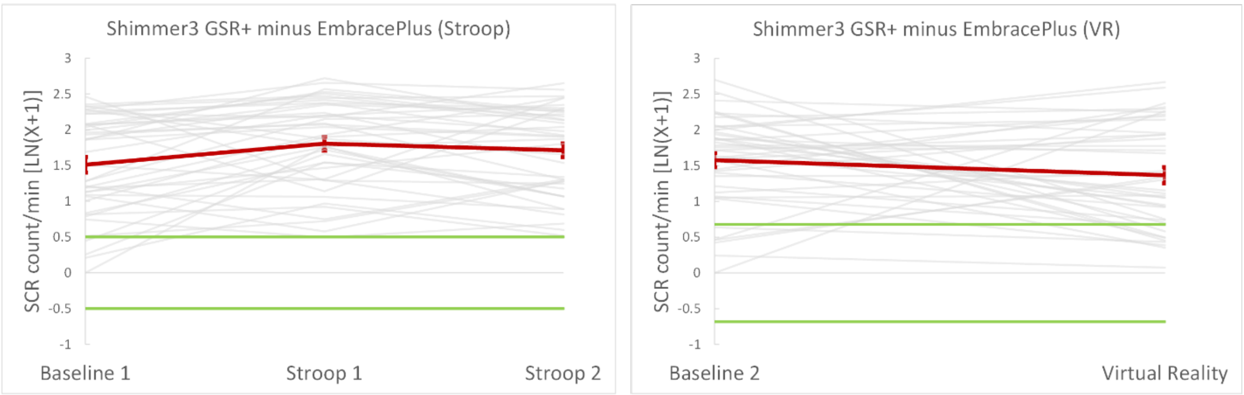

Supplement: Supplementary file 1 [file sensors-26-06795-s001.zip › Supplementary File S7 LN(x+1)-scaled difference plots.pdf]
